# Effects of treadmill running on anxiety- and craniofacial pain-like behaviors with histone H3 acetylation in the brain of mice subjected to social defeat stress

**DOI:** 10.1371/journal.pone.0318292

**Published:** 2025-01-27

**Authors:** Kajita Piriyaprasath, Mana Hasegawa, Yuya Iwamoto, Rantaro Kamimura, Andi Sitti Hajrah Yusuf, Noritaka Fujii, Kensuke Yamamura, Keiichiro Okamoto

**Affiliations:** 1 Division of Oral Physiology, Faculty of Dentistry, Niigata University Graduate School of Medical and Dental Sciences, Niigata, Japan; 2 Department of Restorative Dentistry, Faculty of Dentistry, Naresuan University, Phitsanulok, Thailand; 3 Division of General Dentistry and Dental Clinical Education Unit, Niigata University Medical and Dental Hospital, Niigata, Japan; 4 Division of Dental Clinical Education, Faculty of Dentistry, Niigata University Graduate School of Medical and Dental Sciences, Niigata, Japan; 5 Division of Orthodontics, Faculty of Dentistry, Niigata University Graduate School of Medical and Dental Sciences, Niigata, Japan; 6 Department of Oral and Maxillofacial Surgery, Faculty of Dentistry, Hasanuddin University, Makassar, Indonesia; 7 Sakeology Center, Niigata University, Niigata, Japan; Tokai University, School of Medicine, JAPAN

## Abstract

This study examined the effects of treadmill running (TR) regimens on craniofacial pain- and anxiety-like behaviors, as well as their effects on neural changes in specific brain regions of male mice subjected to repeated social defeat stress (SDS) for 10 days. Behavioral and immunohistochemical experiments were conducted to evaluate the impact of TR regimens on SDS-related those behaviors, as well as epigenetic and neural activity markers in the anterior cingulate cortex (ACC), insular cortex (IC), rostral ventromedial medulla (RVM), and cervical spinal dorsal horn (C2). Behavioral responses were quantified using multiple tests, while immunohistochemistry measured histone H3 acetylation, histone deacetylases (HDAC1, HDAC2), and neural activity markers (FosB and phosphorylated cAMP response element-binding protein (pCREB). The effects of both short-term TR (2 days, TR2) and long-term TR (10 days, TR10) regimens were conducted. TR10 significantly reduced anxiety- and formalin-evoked craniofacial pain-like behaviors in SDS mice. It normalized SDS-induced increases in histone H3 acetylation in both the anterior and posterior portions of the ACC, as well as the anterior portion of the IC. These inhibitory effects were also observed in SDS-related increases in HDAC1, FosB, and pCREB expression. Additionally, TR10 normalized increased histone H3 acetylation in the RVM and C2 regions, with specific effects on FosB and pCREB expression observed in the C2 region. In contrast, TR2 showed limited effects on craniofacial pain-like behaviors but reduced anxiety-like behaviors in SDS mice. Under sham conditions, TR2 had minimal impact on histone H3 acetylation. Paradoxically, TR2 increased formalin-evoked craniofacial pain-like behaviors during the early phase despite not altering acetylated histone H3 expression. In conclusion, the TR10 regimen is effective in attenuating SDS-induced craniofacial pain- and anxiety-like behaviors, likely by normalizing epigenetic modifications and neural activity in key brain regions.

## Introduction

A growing body of evidence demonstrates that psychophysical stress can exacerbate chronic pain in the craniofacial region, particularly in conditions like chronic temporomandibular disorders (TMDs) [1–3]. Both clinical and preclinical studies have revealed functional changes in specific brain regions associated with psychological stress, suggesting potential mechanisms underlying the increased craniofacial pain [4–8]. Traditional strategies for managing chronic craniofacial pain have relied on conservative approaches [1, 2]. However, in recent years, there has been increasing interest in non-pharmacological interventions for managing chronic pain [9–11]. Among these, particularly in the preclinical studies, various forms of physical activities—whether voluntary, like wheel running, or involuntary, like treadmill running (TR)—have demonstrated significant benefits. These include reductions in chronic pain, as well as alleviation of anxiety and depression, which often accompany chronic pain and amplify pain perception [12, 13].

The TR regimen is frequently utilized due to its advantages, including customizable parameters such as intensity and duration, offering greater control compared to other forms of exercise, such as voluntary exercise [11]. While TR is a form of forced physical activity, it can attenuate psychophysical stress-induced behavioral and neural responses [14]. Previously, we reported that 10 days of daily TR reduced craniofacial pain-like behaviors and neural activities in the medullary dorsal horn (C2) region of mice subjected to repeated social defeat stress (SDS) [7]. This helps alleviate increased anxiety and chronic pain associated with psychophysical stressors [15, 16].

Epigenetic changes are recognized as a basis for chronic disorders. Recently, changes in the level of histone H3 acetylation, a crucial process in epigenetic changes, play a crucial role in behavioral alterations to conditions such as anxiety and chronic pain [17–20]. Furthermore, histone deacetylation, mediated by histone deacetylases (HDACs), is a regulatory mechanism of epigenetic changes involved in chronic disorders [21]. While the link between HDACs and psychological distress [22] or nociception [19, 23] varies among reports, HDAC inhibition is shown to reduce anxiety and chronic pain by modulating histone H3 acetylation in the brain [24, 25]. Among several classes of HDACs, HDAC 1 and 2 have been demonstrated to regulate anxio-depression [26] and nociception [17, 19, 27] in various psychophysical stress and pain models.

This study evaluated the modulatory effects of TR on the expressions of histone H3 acetylation, HDAC1, and HDAC2 in the brain, including the anterior cingulate cortex (ACC) and insular cortex (IC) from the brain cortices and the rostral ventromedial medulla (RVM) and upper cervical spinal dorsal horn (C2). While neural changes seen in the ACC and IC are documented to regulate anxiety and nociception, those in the RVM and C2 areas, as components of descending pain controls, have been linked to increased nociception [28]. Indeed, our previous findings supported these notions that mice subjected to repeated SDS for 10 days increased the expression for markers for neural activities, such as c-Fos and FosB immunoreactivities, in these areas [5].

In this study, we hypothesize that a 10-day TR (TR10) regimen could normalize SDS-induced alterations of histone H3 acetylation and HDAC1 and HDAC2 expression in several areas of the brain. Additionally, we assessed the potential effects of a 2-day TR (TR2) regimen to determine whether a shorter TR duration produces comparable behavioral and epigenetic changes under SDS conditions, as observed with TR10, in these specific brain regions.

To further clarify the effects of TR on SDS-related neural responses, we also assessed the expression of FosB and phosphorylated cyclic-AMP response element-binding protein (pCREB), which are markers linked to anxiety and chronic pain [29–32]. Changes in these markers have been associated with psychophysical stress-induced behavioral and neural alterations related to anxiety and chronic pain [5, 29, 30]. Moreover, evidence suggests potential connections between these markers and neural functions influenced by psychophysical stress conditions [32]. Based on our hypothesis, TR might serve as a therapeutic strategy to mitigate the exacerbation of craniofacial pain induced by psychophysical stress, potentially by targeting epigenetic mechanisms and modulating neural activity in relevant regions.

## Methods

### 1. Animals

Our study used 150 male C57BL/6J adult mice and eight Institute of Cancer Research (ICR) mice. All animal experimental procedures underwent a thorough review and received approval from the Intramural Animal Care and Veterinary Science Committee of Niigata University (permit number: SA00611) and performed under the Guiding Principles for Care and Use of Laboratory Animals (National Institutes of Health).

On arrival, male mice (C57BL/6J, Charles River Laboratories) were six weeks old and weighed between 20 and 25 g. The mice were housed in clear acrylic cages (30 × 20 × 15 cm) and allowed free access to standard pellet food and water. The C57BL/6J mice (four per cage) were housed in cages for at least one week to facilitate adaptation. The 6-week-old male ICR mice were housed individually. The housing facility was temperature- and humidity-controlled with a 12-hour light/dark cycle (light phase: 7:00–19:00).

### 2. Social defeat stress conditionings

Previous reports provided a detailed description of the social defeat stress (SDS) conditioning procedure [5, 7, 33]. In brief, the aggressive behavior of ICR mice (aggressors) was assessed by placing C57BL/6J mice in individual cages. Aggressors were excluded if they did not initiate a physical attack on the C57BL/6J mice within 2 minutes. C57BL/6J mice were introduced to a different aggressor each day for 10 consecutive days. During this period, C57BL/6J mice were allowed to interact physically with the aggressor for 10 minutes per day, and these mice were classified as SDS mice. Careful observation was conducted to prevent excessive confrontation and physical harm to the SDS mice. No tissue damage or bleeding associated with SDS conditioning was observed. After each 10-minute SDS conditioning, SDS mice were housed with the aggressor for 2 hours, separated by a Plexiglas screen. Control mice, referred to as Sham mice, were just placed in clear acrylic cages adjacent to the aggressor’s cage for 10 minutes daily and handled similarly to the SDS mice throughout the experimental schedule. After this 10-minute treatment, the cages of Sham mice were returned to separate areas where the Sham mice could not see the aggressor cages. Sham mice never witnessed SDS mice undergoing SDS conditioning.

### 3. Experimental design

**Fig 1:**
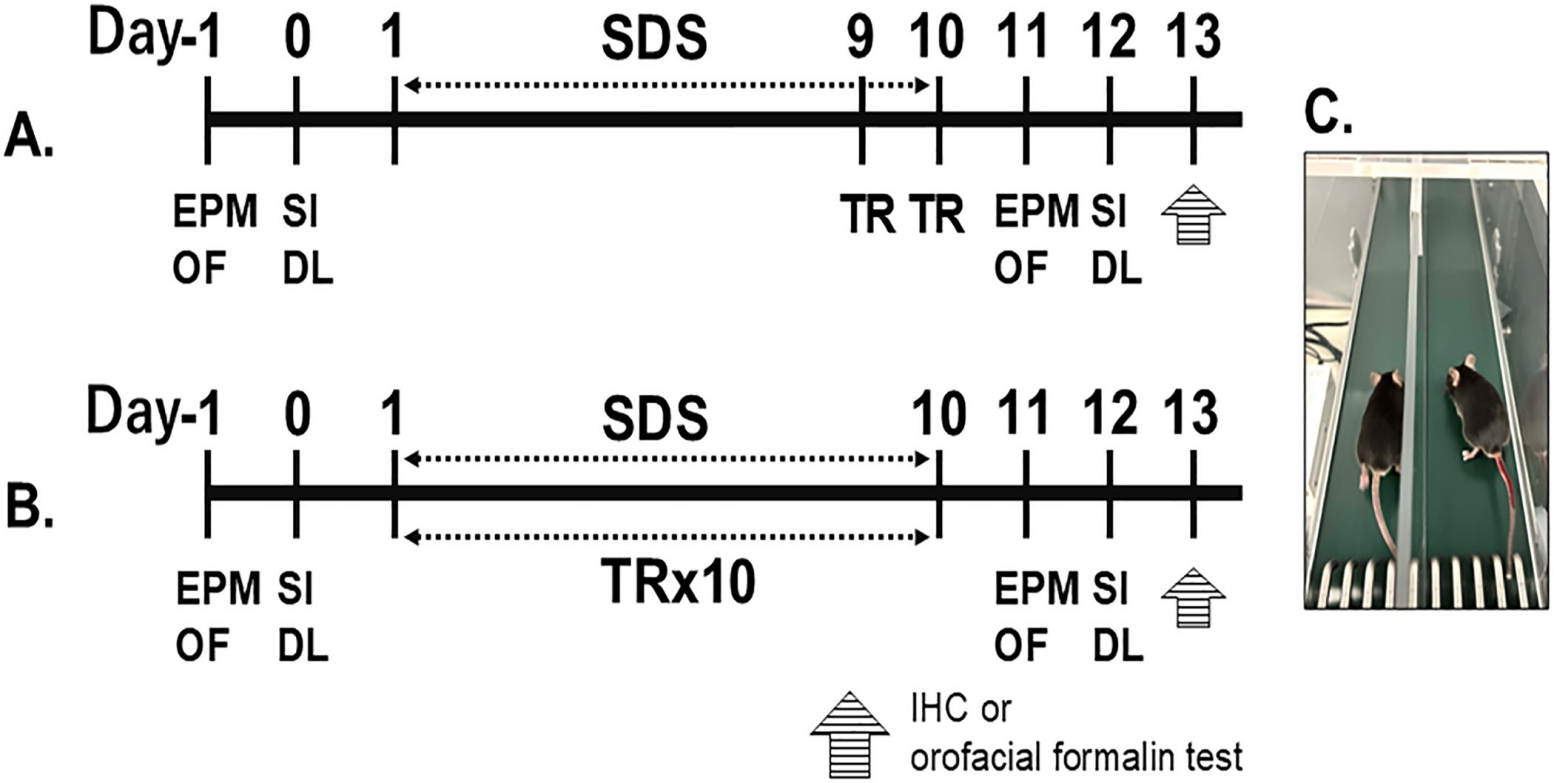
The diagram outlines the experimental design. **Experiment 1 (A):** Mice were exposed to treadmill running (TR) or remained sedentary on Days 9 and 10, following the daily Sham or SDS conditioning sessions. **Experiment 2 (B):** Mice participated in daily TR or sedentary sessions from Day 1 through Day 10 in Sham and SDS mice. (**C)** The image shows the motorized rodent treadmill used for TR. The detailed sample size of each experiment was presented in the S1 File. Abbreviations: IHC, immunohistochemistry.

**Fig 2:**
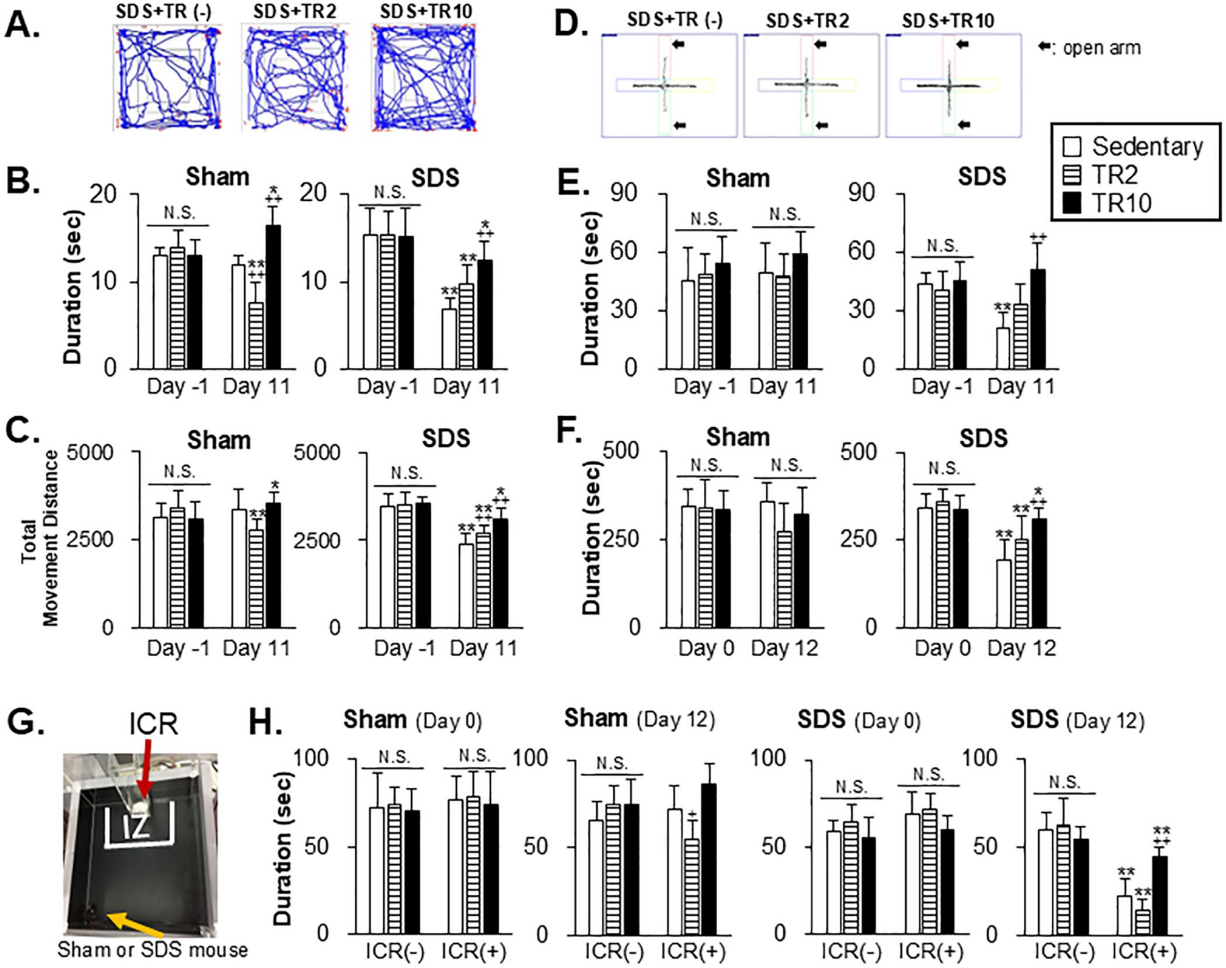
Effect of treadmill running on anxiety-like behavior. (**A**) Representative tracking routes of mice during the Open Field (OF) test. The images depict the movement patterns of SDS mice within the OF. (**B**) Data summary for the effect of sedentary and treadmill running (TR) sessions on time spent in the center area during the OF test. (**C**) Data summary of the effect of sedentary and TR sessions on total movement distance in the OF during the OF test. (**D**) Representative tracking routes of mice during the Elevated Plus Maze (EPM) test. The images depict the movement patterns of SDS mice within the EPM, illustrating their exploration of the open and closed arms. (**E**) Data summary of the effect of TR on time spent in the open arms in Sham (left panel) and SDS (right panel) mice during the EPM test. (**F**) Data summary of the effect of TR on time spent in the light chamber in Sham (left panel) and SDS (right panel) mice during the Dark and light box (DL) test. (**G**) The image of the social interaction (SI) field, delineated with the interaction zone (IZ) employed in the SI test, is depicted. (**H**) Data summary of the effect of TR sessions on time spent in the IZ during the SI test on Day 0 and Day 12. *,** versus before (Day -1 or Day 0) Sham or SDS conditionings (**B**, **C**, **E**, and **F**) or ICR (-) (**H**) in the corresponding TR sessions. +, ++ versus the sedentary group on the corresponding Day (**B**, **C**, **E**, and **F**) or ICR (+) session (**H**). *, + *p* < 0.05, **, ++ *p* < 0.001.

Two experimental protocols were conducted. In Experiments 1 (Fig 1A) and 2 (Fig 1B), C57BL/6J mice were divided into two groups and underwent either SDS (SDS mice, n = 74) or sham (sham mice, n = 76) conditioning for 10 days. Mice in each conditioning group were further divided into subgroups to undergo either sedentary or treadmill running (TR) conditionings for 2 days (TR2) or 10 days (TR10).

Anxiety-like behaviors were assessed before and after the SDS (n = 36) or sham (n = 48) conditioning, with or without TR, using the elevated plus maze (EPM), open field (OF), dark and light box (DL), and social interaction (SI) tests. These groups were employed for the further immunohistochemical experiments. Additionally, separate groups of animals were used to evaluate the effects of TRs on craniofacial pain-like behaviors in SDS (n = 38) and sham (n = 28) mice. To minimize experimental bias, the experimenters who conducted the behavioral and immunohistochemical analyses were not involved in developing the sham or SDS conditioning or TR sessions. The sample size was presented in detail in the S1 File.

### 4. Measurements of anxiety-like behaviors

Multiple behavioral procedures were conducted to assess anxiety-like behaviors before (Day -1 or Day 0) and after (Day 11 or Day 12) experiment protocol in each group (Fig 1), and results were compared between groups. The behavioral procedures used were identical to those described in our previous studies [5, 7, 34]. The mice were acclimated to the testing room for 1 h before starting the test. The experimenter was blinded to all treatments.

#### Open Field (OF) test

A mouse was placed in a clear acrylic cage with black frosting Plexiglas floor (45 x 45 x 30 cm) for 5 mins. The time spent in the center zone and total movement distances in the OF were measured using digital counters with infrared sensors (SCANET MV-40 MOV, MELQUEST Co., Ltd., Toyama, Japan).

#### Elevated plus-maze (EPM) test

A mouse was placed in the center of the maze that consisted of two opened and two closed arms (PM-DR25M-S, Brain Science Idea. Co., Ltd., Osaka, Japan). The maze was raised around 50 cm above the ground. Time spent in the open arms was recorded over a 5-minute period for subsequent analysis.

#### Dark and Light box (DL) test

A mouse was placed in a dark and light box with two compartments (14 x 14 x 14 cm), one light chamber and the other dark chamber, which were joined by the opening. Mice were initially placed in the light chamber, and the behavior of the mouse was observed for 15 minutes. The time spent in the light chamber was measured by digital counters with infrared sensors (SCANET MV-40, MELQUEST, Toyama, Japan).

#### Social Interaction (SI) test

The procedure for the SI test was described in our previous report [5]. Each mouse was placed in the center of an OF arena of a transparent acrylic cage with a black frosting Plexiglas floor (45 × 45 × 30 cm). The OF was separated into two zones of interest: the interaction zone (IZ) and the corner zone. This study evaluated the time spent in the IZ for 2.5 min in the absence and presence of the aggressor (ICR) mouse using digital counters with infrared sensors (SCANET MV-40, MELQUEST, Toyama, Japan).

### 5. Treadmill running (TR)

In Experiment 1 (Fig 1A), mice underwent either sedentary or TR sessions from Day 9 to Day 10 (TR2) 30 min after daily Sham or SDS conditionings. In Experiment 2 (Fig 1B), TR sessions were conducted from Day 1 to Day 10 (TR10). The TR procedure was described in our previous report [7]. Briefly, TR was involved in running at 6 meters per minute (m/min) for 30 minutes with no inclination on a motorized rodent treadmill (TMS-2B, MELQUEST Co., Ltd., Toyama, Japan). The treadmill featured a two-lane design with a single belt and a dividing wall above the tread surface (Fig 1C). Sedentary mice were placed in the treadmill apparatus for 30 minutes without running. After each session, the treadmill was cleaned with 70% ethanol, wiped, and air-dried before the next use.

### 6. Measurement of craniofacial nociception

#### Orofacial formalin behavioral test

**Fig 3:**
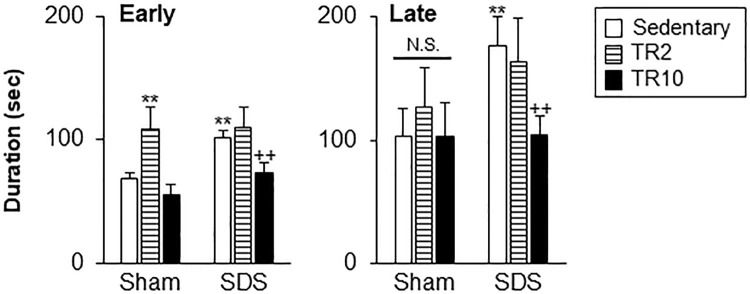
Effect of treadmill running on formalin-evoked craniofacial pain-like behav iors. ** versus Sham–Sedentary. ++ versus SDS–Sedentary. N.S. no significant when compared to the sedentary group. **, ++ *p* < 0.001. In the early phase (Fig 3, left panel), the time spent on craniofacial pain-like behaviors in SDS mice with sedentary sessions was significantly greater than in Sham mice with sedentary sessions (*p* < 0.001). In the Sham group, time spent on these behaviors after TR2 sessions was significantly greater than in Sham mice with sedentary sessions (*p* < 0.001). In the SDS group, these behavioral times in TR10 group were significantly smaller than those in sedentary sessions (*p* < 0.001).

The orofacial formalin test was performed on Day 13 to measure craniofacial pain-like behaviors (Fig 1) [5, 7]. In brief, 2 μL of 2.5% formalin (in 0.9% saline) was injected into the mid-region of the left masseter muscle using a Hamilton syringe. The animals were gently restrained with a soft plastic net for less than 5 seconds during the injection. Afterward, the mice were placed back into the observation cage, and the duration of grooming or rubbing behavior on the ipsilateral side was recorded. Cumulative time spent on these behaviors was measured using a stopwatch in 10 successive 3-minute intervals. The results were analyzed for cumulative behavioral time during the early (0–9 minutes) and late (12–30 minutes) phases and were shown in Fig 3. The experimenter was blinded to all treatments. The sample size was presented in detail in the S1 File.

### 7. Immunohistochemistry (IHC)

The effects of TR on various immunoreactivities were assessed in Sham and SDS mice on Day 13 (Fig 1). The IHC experiments were conducted using the same groups of animals that were evaluated for anxiety-like behaviors. The sample size in detail was presented in the S1 File.

#### Animal preparations

After the completion of behavioral experiments, employed to assess the conditions of anxiety- but not formalin-evoked craniofacial pain, mice were randomly selected for the IHC on Day 13. Mice were deeply anesthetized with three mixed agents that were prepared with 0.3 mg/kg of medetomidine (Dosmitor; Nippon Zenyaku Kogyo), 4.0 mg/kg of midazolam (Midazolam; Sandoz), and 5.0 mg/kg of butorphanol (Vetorphale; Meiji Seika Pharma) 24 h after the last stress conditionings. Mice were perfused through the heart with saline (20 ml), followed by cold 4% paraformaldehyde in 0.1M phosphate buffer saline (PBS, pH = 7.4, 20 ml). The brains, including the anterior cingulate cortex (ACC), insular cortex (IC), rostral ventromedial medulla (RVM), and upper cervical spinal cord (C2), were removed and post-fixed in 4% paraformaldehyde for a few days and stored in sucrose (30% in 0.1M PBS) at 4°C overnight. Those were cut into transverse sections (0.04 mm thick) using a freezing microtome (REM-710, RETRATOME, YAMATO, Saitama, Japan). Sections were collected in five wells containing cold 0.1 M PBS and transferred serially to multi-well tissue cultures that contained cold 0.1M PBS and were used for IHC.

#### Immunostaining

After the sections were rinsed with PBS several times, they were incubated in affinity-purified rabbit acetylated (acetyl) histone H3 polyclonal antibody (1:2000, Sigma-Aldrich, Massachusetts, USA), HDAC1 polyclonal antibody (1:5000, Abcam, Cambridge, UK), HDAC2 monoclonal antibody (1:5000, Abcam, Cambridge, UK), FosB monoclonal antibody (1:2000, Abcam, Cambridge, UK), or phospho-CREB polyclonal antibody (1:5000, Sigma-Aldrich, Massachusetts, USA) in PBS with Tween 20 (PBST) and 5% normal goat serum overnight at room temperature. Following a double rinse with PBS, the sections were treated with biotinylated goat anti-rabbit IgG antibody (1:200, Vector Laboratories, Burlingame, CA, USA) and 5% NGS in PBST for 2 hours at room temperature. After washing sections with PBS twice, an avidin-biotin-peroxidase complex (Vector Laboratories, USA) was added to the PBS for 1 hour. Sections were washed with Tris-buffered saline (TBS) twice and then incubated in diaminobenzidine and nickel solution activated by 0.01% peroxidase. After visualizing immunoreactivity, all sections were washed with TBS, mounted on the glass slides, and dried. Then, sections on the glass slides were dehydrated in ethanol, cleared in xylene, and then cover-slipped. Specific immunostaining for each primary antibody was not identified after the omission of it.

#### Quantifications

Sections were identified based on landmark structures at each level using the Franklin and Paxinos atlas (3rd edition) [35], with reference points corresponding to specific distances rostral to bregma: anterior portion of the ACC (+1.34 mm), posterior portion of the ACC (+1.10 mm), anterior portion of the IC (+1.34 mm), and posterior portion of the IC (-0.58 mm). The locations of these portions of the ACC and IC were selected for analysis due to several considerations. First, each region has been shown to play distinct roles in the regulation of nociception and emotion [36–38]. Second, while both the ACC and IC are involved in pain perception, neural activity in these regions is positively correlated with the intensity of pain perception [39]. However, the precise roles of each region remain incompletely understood. The locations of the RVM (-6 to -6.2 mm from bregma) and C2 (-4 to -5 mm rostral to the obex) regions analyzed in this study were described in our previous reports [5, 7]. In this study, the RVM included several areas, such as the raphe magnus nucleus, nucleus paragigantocellularis reticularis pars alpha (GiA), and part of the lateral paragigantocellular reticular nucleus. The C2 region, located caudal to the medulla, was analyzed for cell quantifications within its superficial and deep laminae of the dorsal horn. Images of each section were taken under the same conditions and converted to an eight-bit grayscale.

Two or three sections were randomly selected. Cell counts were conducted bilaterally in the outlined area in the anterior portion of ACC (0.3 x 0.6 mm), posterior portion of ACC (0.3 x 0.5 mm), the anterior and posterior portion of IC (0.6 x 0.5 mm), RVM (1.0 x 0.3 mm), and C2 (0.5 x 0.3 mm) using Image J (National Institutes of Health, USA). The results represent the average number of cells calculated from the left sides of the ACC and IC and that from the left side of the C2 region because the number of immuno-positive cells on the left side was similar to that on the right side in each stress group.

#### Statistical analysis

Statistical analyses for the behavioral and IHC experiments were conducted using SPSS Statistics (version 21.0; IBM). Behavioral data were analyzed using a two-way repeated-measures analysis of variance (ANOVA). The analysis focused on two main factors: the within-subject factor, which examined the effects of TR on behaviors before and after SDS, and the between-subject factor, which compared the impact of different stress treatments (Sham vs. SDS) with or without TR administration. The IHC experiments were assessed by one-way ANOVA. Post hoc tests comprising individual comparisons were performed using the Bonferroni test. The results are presented as the average ± standard deviation (SD). Spearman’s test was conducted to assess the correlation between craniofacial pain-like behaviors versus the number of acetyl histone H3-positive cells. Time spent on formalin-evoked licking behaviors for 30 min observation was compared with the number of positive cells in the ACC, IC, RVM, and C2 regions. A statistical level of *p* < 0.05 was considered significant.

## Results

### 1. Effect of TR on anxiety-like behaviors

#### Open field test

Fig 2A illustrates the examples demonstrating the trajectory of a mouse subjected to repeated SDS with sedentary, TR2, or TR10 sessions moving in the open field (OF). Fig 2B and 2C summarized the results of time spent in the center area and total movement distance in the OF, respectively.

Significant main effects of Sham and SDS conditioning were observed for the time spent in the center area (Sham: F(1, 29) = 10.1, *p* = 0.003; SDS: F(1, 28) = 120.2, *p* < 0.0001) and between groups (Sham: F(2, 29) = 15.8, *p* < 0.0001; SDS: F(2, 28) = 4.4, *p* = 0.002) as measured in Fig 2B. Additionally, significant main effects of Sham and SDS conditioning were found for total movement distance in the open field (OF) test (Sham: F(1, 29) = 3.5, *p* = 0.01; SDS: F(1, 28) = 120.3, *p* < 0.0001), as well as significant between-group effects (Sham: F(2, 29) = 3.5, *p* = 0.01; SDS: F(2, 28) = 4.78, *p* < 0.0001). Post hoc analysis using the Bonferroni test revealed the following.

On Day -1 prior to Sham or SDS conditionings with or without TRs, both the time spent in the center area and total movement distance in the open field were similar across groups (p > 0.05).

On Day 11, after TR sessions in Sham mice, the time spent in the center area and total movement distance in the OF in TR2 and TR10 were significantly decreased (P < 0.001) and increased (*p* < 0.05), respectively, compared to Day -1. Additionally, on Day 11, the time spent in the center area in TR2 and TR10 was lower and higher, respectively, than in sedentary mice (Fig 2B, *p* < 0.001).

In SDS mice (Fig 2B and 2C), time spent in the center area and total movement distance in sedentary, TR2, and TR10 groups on Day 11 were significantly decreased compared to Day -1. However, On Day 11, these parameters seen in SDS mice subjected to TR10 were significantly greater than in SDS mice subjected to sedentary sessions. The total movement distance in SDS mice subjected to TR2 was significantly greater than in SDS mice subjected to sedentary sessions (*p* <0.001).

#### Elevated plus maze test

Fig 2D illustrates the examples demonstrating the trajectory of a mouse subjected to repeated SDS with sedentary, TR2, or TR10 sessions moving in the elevated plus maze. A significant main effect of SDS conditioning was observed (F(1, 28) = 242, *p* < 0.0001), along with a significant effect between groups (F(2, 28) = 9.7, *p* < 0.0001) for the time spent within the open arms measured in the SDS group (Fig 2F). No main effect was observed in the Sham group. Post hoc analysis using the Bonferroni test revealed the following.

In Sham mice, time spent in open arms on Day-1 and Day 11 was similar across groups (Fig 2E, *p* > 0.05). In SDS mice, time spent in open arms in sedentary mice on Day 11 was significantly decreased compared to Day -1 (Fig 2E, *p* < 0.001). Furthermore, On Day 11, time spent in open arms in the SDS mice with TR10 was significantly greater than in sedentary mice (*p* < 0.001).

#### Dark and light box test

A significant main effect of SDS conditioning was observed (F(1, 28) = 222, *p* < 0.0001), along with a significant effect between groups (F(2, 28) = 7.4, *p* < 0.0001) for behaviors measured in the SDS group (Fig 2F). No main effect was observed in the Sham group. Post hoc analysis using the Bonferroni test revealed the following.

In Sham mice (Fig 2F), the time spent in the light chamber on Day 0 and Day 12 was similar across all groups (*p* > 0.05). In SDS mice (Fig 2F), the time spent in the light chamber in all groups on Day 12 was significantly decreased compared to Day 0 (*p* < 0.05). However, on Day 12, time spent in the light chamber in SDS mice with TR10 was significantly greater than that in sedentary mice (*p* < 0.001).

#### Social interaction test

Fig 2G illustrates the social interaction (SI) arena, highlighting the interaction zone (IZ), where the time spent in the IZ was compared between groups.

A significant main effect was observed among the three groups (F(2, 28) = 10.2, *p* < 0.001) and for the presence of the ICR mouse (F(1, 28) = 111.7, *p* < 0.0001) in the behaviors measured on Day 12 in SDS group (Fig 2H). No main effects were observed in the Sham group. Post hoc analysis using the Bonferroni test revealed the following.

On Day 0, the time spent in the IZ was similar across groups in both Sham (Fig 2H, left two panels) and SDS (Fig 2H, right two panels) mice, regardless of whether the ICR mouse was present or absent (*p* > 0.05). On Day 12, in Sham mice exposed to the aggressor (the ICR mouse), TR10 had no significant effect on the time spent in the IZ compared to Sham mice with sedentary sessions (*p* > 0.05). However, in TR2 mice exposed to the aggressor, the time spent in the IZ was significantly lower than in Sham mice with sedentary sessions (*P* < 0.05).

In SDS mice, on Day 12, the time spent in the IZ in the presence of the aggressor was significantly reduced in all groups compared to when the aggressor was absent (*p* < 0.001). However, in the presence of the aggressor, SDS mice with TR10 sessions spent significantly more time in the IZ compared to sedentary mice (*p* < 0.001).

### 2. Effect of TR on formalin-evoked craniofacial pain-like behaviors

A significant main effect of the six treatment groups was observed for behavioral responses during both the early (F(5, 62) = 33.66, *p* < 0.0001) and late (F(5, 62) = 15.5, *p* < 0.0001) phases (Fig 3). Post hoc analysis using the Bonferroni test revealed the following results.

In the late phase (Fig 3, right panel), the time spent on craniofacial pain-like behaviors in SDS mice with sedentary sessions was significantly greater than in Sham mice with sedentary sessions (*p* < 0.001). In Sham mice, TR has no effect (*p* > 0.1) on behaviors. In SDS mice, time spent on craniofacial pain-like behaviors in the TR10 group was significantly smaller than in the sedentary group (*p* < 0.001).

### 3. Immunohistochemistry

#### Effect of TR on acetylated histone H3, HDAC1, and HDAC2 expression in the ACC and IC regions

**Fig 4:**
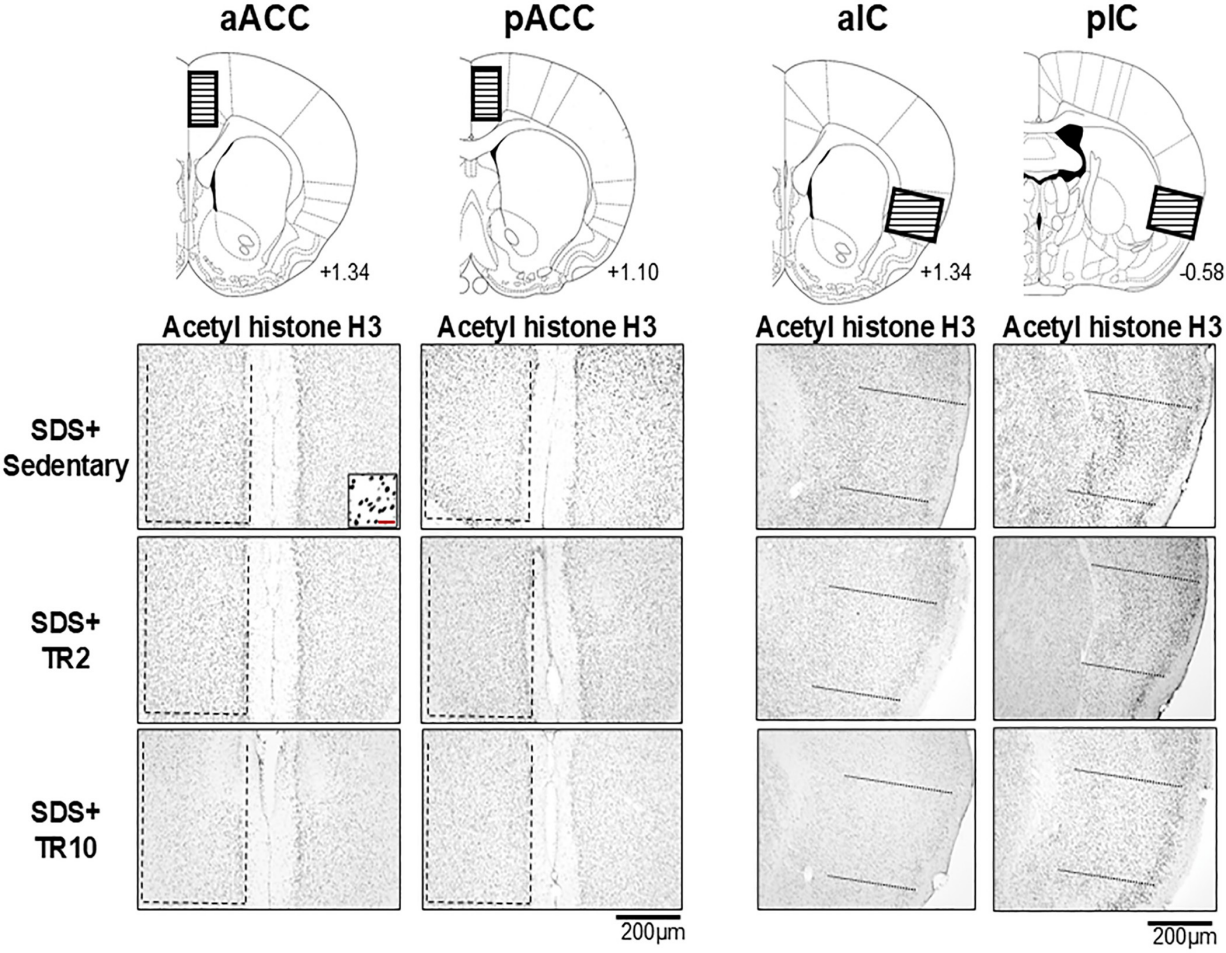
Microphotographs displaying acetylated (acetyl) histone H3 immunoreactivity in SDS mice within the anterior and posterior portions of the ACC (aACC and pACC) and IC (aIC and pIC). Cell counts were conducted within the boxed areas highlighted in each region, as illustrated in the brain schema shown in the top panels. The number below each coronal section indicates the rostrocaudal distance from the bregma (in mm). Dashed lines delineate the borders of the evaluated regions. The scale bar in the insets represents 50 μm.

**Fig 5:**
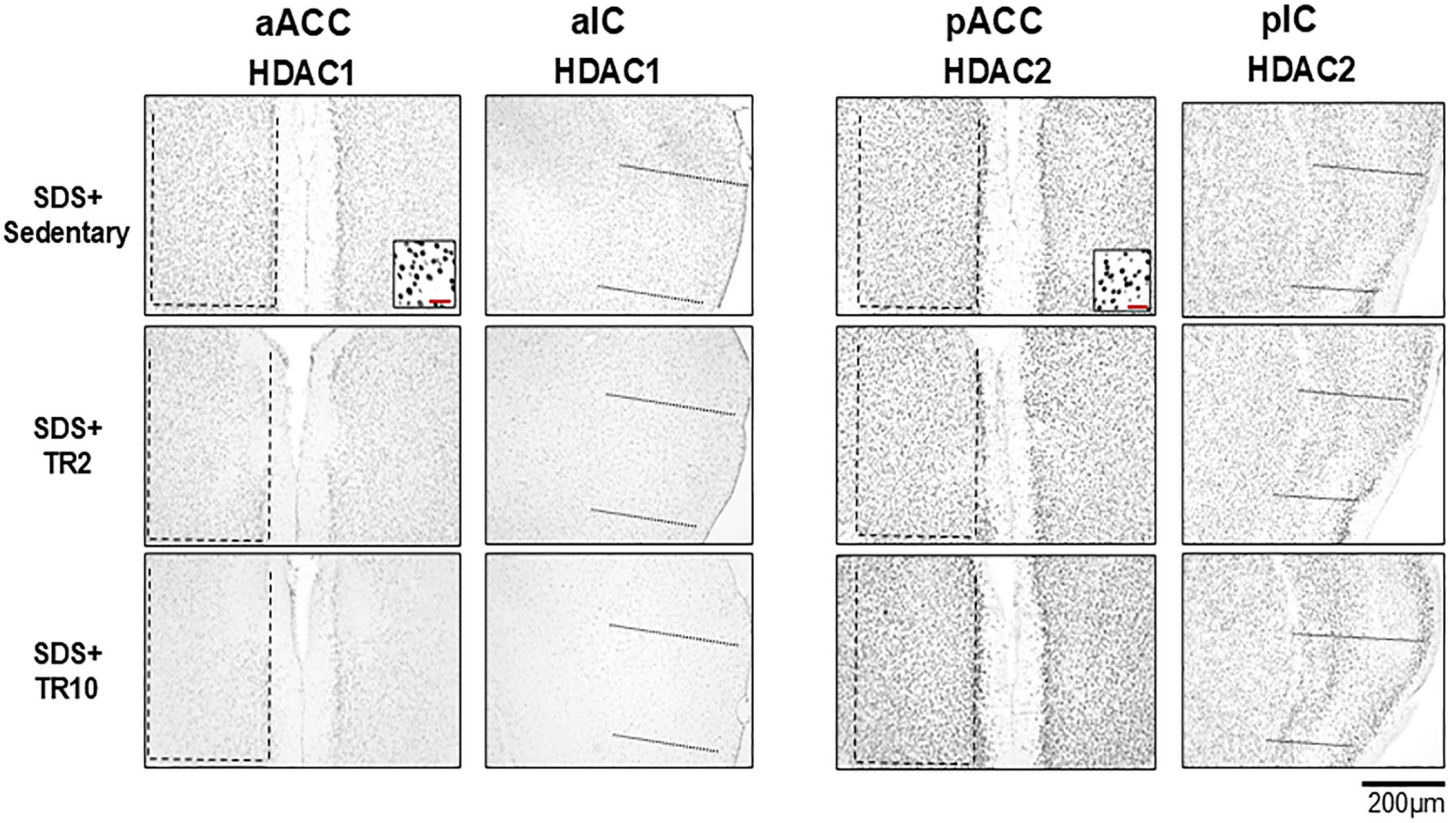
Microphotographs displaying HDAC1 and HDAC2 immunoreactivities in SDS mice in the anterior and posterior portions of the ACC (aACC and pACC) and IC (aIC and pIC). Cell counts were conducted within the boxed areas highlighted in each region, as illustrated in the brain schema shown in Fig 4. The scale bar in the insets represents 50 μm.

Figs 4 and 5 illustrate examples of acetylated (acetyl) histone H3, histone deacetylase 1 (HDAC1), and histone deacetylase 2 (HDAC2) immunoreactivities in SDS mice, focusing on the anterior and posterior portions of the ACC and IC. The areas analyzed quantitatively are indicated by hatched and dotted boxes in the schematic at the top of Fig 4. These immunoreactivities are predominantly localized in the cell nuclei, as observed in the high-magnification images of Figs 4 and 5 (insets).

The ANOVA revealed the significant main effect of the number of histone H3 acetylation and HDAC1-positive cells across six groups in the anterior (histone H3 acetylation: F(5, 40) = 43.0, *p* < 0.001, HDAC1: F(5, 32 = 10.4, *p* < 0.0001) and posterior (histone H3 acetylation: F(5, 43) = 21.7, *p* < 0.001, HDAC1: F(5, 28 = 7.9, *p* < 0.0001) portions of ACC. Similarly, significant effects were observed in the anterior (histone H3 acetylation: F(5, 39) = 16.5, *p* < 0.001, HDAC1: F(5, 28 = 15.4, *p* < 0.0001) and posterior (histone H3 acetylation: F(5, 42) = 9.0, P < 0.001, HDAC1: F(5, 28 = 14.2, *p* < 0.0001) portions of IC. In contrast, HDAC2 expression showed no significant main effects in the anterior or posterior portion of the ACC. However, significant main effects were observed for HDAC2 in the anterior (F(5, 38) = 2.9, *p* < 0.024) and posterior (F(5, 37) = 8.3, *p* < 0.0001) portions of the IC. Post hoc analysis using the Bonferroni test revealed the following.

**Fig 6:**
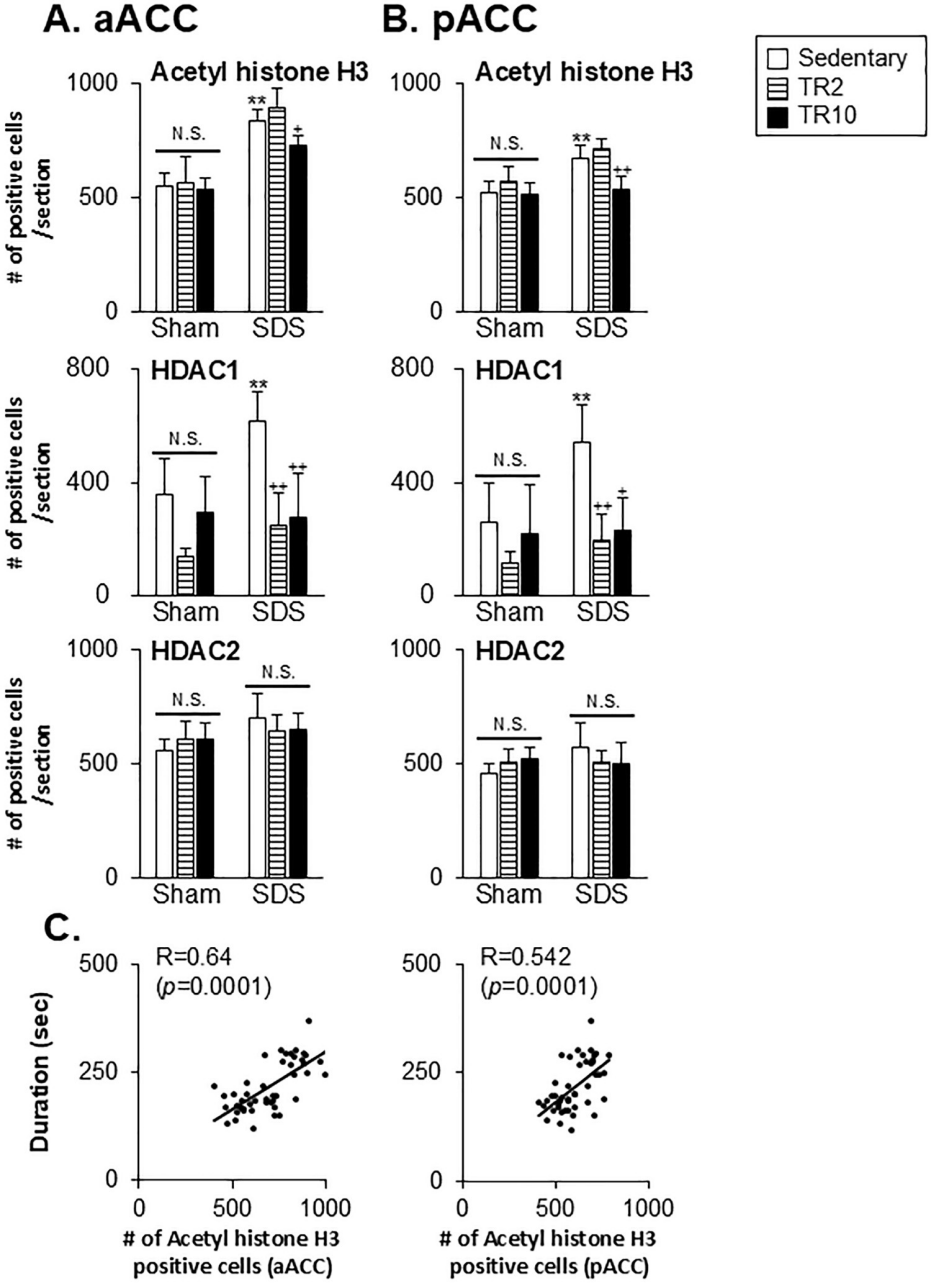
Data summary of the effect of treadmill running on acetylated (acetyl) histone H3, HDAC1, and HDAC2 expression in the anterior (**A**; aACC) and posterior (**B**; pACC) portions of ACC. (**C**) Scatter plots display the correlation between orofacial formalin behavior and acetylated histone H3 expression in the ACC. ** versus Sham–Sedentary group. +, ++ versus SDS-Sedentary group. **, ++ *p* < 0.001. + *p* <0.05. N.S. no significant when compared to the sedentary group.

**Fig 7:**
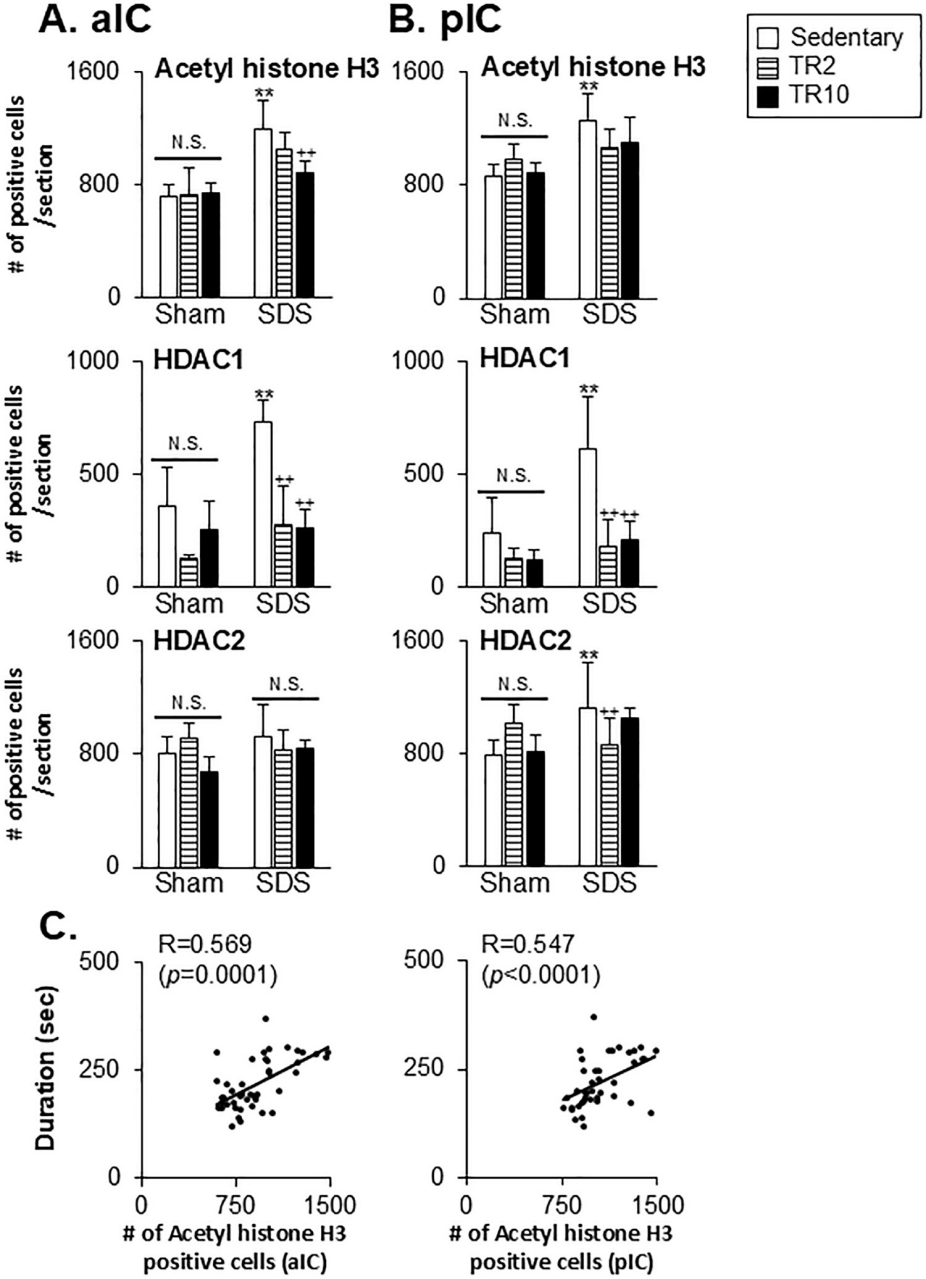
Data summary of the effect of treadmill running on acetylated (acetyl) histone H3, HDAC1, and HDAC2 expression in the anterior portion of IC (**A**; aIC) and posterior portion of IC (**B**; pIC). (**C**) Scatter plots display the correlation between orofacial formalin behavior and acetylated histone H3 expression in the IC. ** versus Sham–Sedentary group. ++ versus SDS-Sedentary group. **, ++ *p* < 0.001. N.S. no significant when compared to the sedentary group.

In SDS mice under sedentary conditions, the number of acetyl histone H3 and HDAC1-positive cells was significantly greater in the anterior portion of ACC (Fig 6A), posterior portion of ACC (Fig 6B), anterior portion of IC (Fig 7A), and posterior portion of IC (Fig 7B) compared to Sham mice (*p* < 0.001). The number of HDAC2-positive cells was significantly higher in the posterior portion of the IC (*p* < 0.001, Fig 7B) but not in the ACC or anterior portion of the IC.

In Sham mice, TR2 or TR10 sessions did not significantly alter the number of acetyl histone H3-positive cells in any region (Figs 6A, 6B, 7A, 7B). Similarly, TR2 and TR10 sessions had minimal effects on HDAC1 and HDAC2 expression in these regions.

In SDS mice, the number of acetyl histone H3 positive cells in TR10 sessions was significantly lower in the anterior portion of ACC (Fig 6A, *p* < 0.05), posterior portion of ACC (Fig 6B, *p* < 0.001), and the anterior portion of IC (Fig 7A, *p* < 0.001) compared to sedentary sessions. However, TR10 displayed less effect on acetyl histone H3 in the posterior portion of IC (Fig 7B, *p* > 0.1). TR2 sessions had less effect in these areas in SDS mice (Figs 6 and 7). Furthermore, in SDS mice subjected to TR2 and TR10 sessions, the number of HDAC1 positive cells across all four regions was significantly lower compared to sedentary sessions (Figs 6 and 7). However, the impact of TR2 and TR10 sessions on HDAC2 immunoreactivities was less pronounced, except in the posterior portion of IC (Fig 7B). Notably, TR2 and TR10 sessions had no significant effect on acetyl histone H3 expression in the posterior portion of IC (Fig 7B).

Spearman’s test was used to assess the correlation between formalin-evoked facial pain-like behaviors and acetyl histone H3 expression in the ACC and IC. Figs 6C and 7C show that time spent on craniofacial pain-like behaviors for 30 min was positively correlated with H3 expression in the anterior portion of ACC (r = 0.64, *p* < 0.0001), posterior portion of ACC (r = 0.542, *p* < 0.0001), anterior portion of IC (r = 0.569, *p* < 0.0001), and posterior portion of IC (r = 0.547, *p* < 0.0001).

#### Effect of TR on FosB and pCREB expression in the ACC and IC regions

FosB and pCREB immunoreactivities were quantified in the same portions of ACC and IC in which the markers for epigenetic changes were assessed. These immunoreactivities are predominantly localized in the cell nuclei, which appeared to be consistent with numerous previous reports [5, 38, 40].

Significant main effect of the number of FosB and pCREB-positive cells across six groups in the anterior (FosB: F(5, 44) = 28.9, *p* < 0.0001, pCREB: F(5, 35 = 15.9, *p* < 0.0001) and posterior (FosB: F(5, 44) = 3.9, *p* < 0.005, pCREB: F(5, 37 = 14.8, *p* < 0.0001) portions of ACC. Similarly, significant effects were observed in the anterior (FosB: F(5, 39) = 4.9, *p* < 0.001, pCREB: F(5, 38 = 27.3, *p* < 0.0001) and posterior (FosB: F(5, 39) = 5.7, *p* < 0.0001, pCREB: F(5, 37 = 14.2, *p* < 0.0001) portions of IC. Post hoc analysis using the Bonferroni test revealed the following.

**Fig 8:**
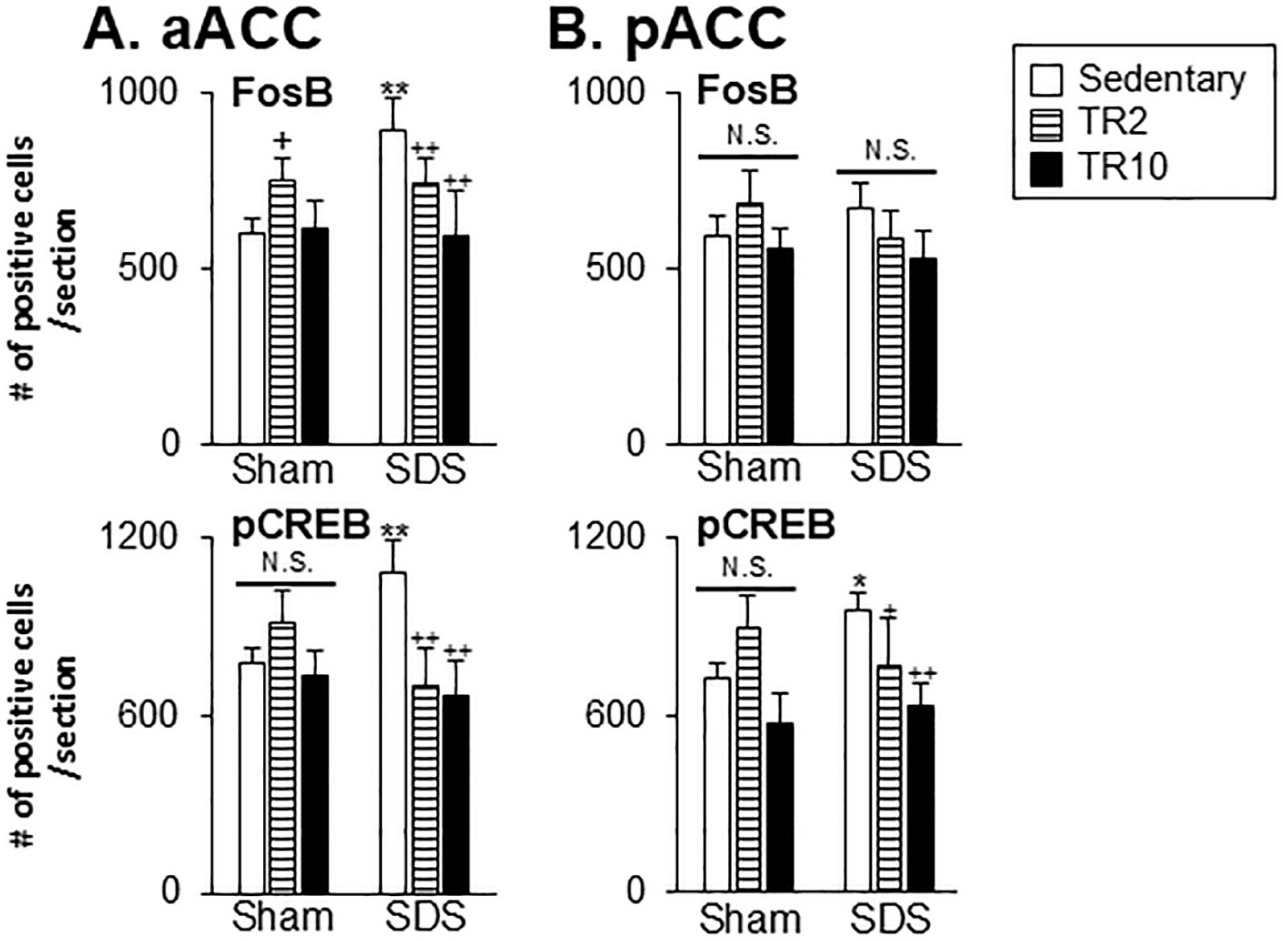
Data summary of the effect of treadmill running on FosB and pCREB expression in the anterior (A; aACC) and posterior (B; pACC) portions of ACC. *, ** versus Sham–Sedentary group. +, ++ versus SDS-Sedentary group. **, ++ *p* < 0.001. *, + *p* < 0.05. N.S. no significant when compared to the Sedentary group.

In the anterior portion of ACC, under sedentary conditions, the number of FosB-positive (*P* < 0.001) and pCREB-positive (*p* < 0.001) cells was significantly higher in SDS mice compared to Sham mice (Fig 8A). In the posterior portion of ACC, SDS mice in sedentary sessions exhibited a significantly greater number of pCREB-positive cells (*p* < 0.05) but not FosB-positive cells (*p* > 0.1), compared to Sham mice in sedentary sessions (Fig 8B).

In Sham mice subjected to TR2 sessions, the number of FosB-positive cells in the anterior portion of ACC was significantly higher compared to Sham mice in sedentary sessions (*p* < 0.05). However, TR2 and TR10 sessions had less effect on FosB and pCREB expression in the posterior portion of ACC, as well as on pCREB expression in the anterior portion of ACC (*p* > 0.05).

In SDS mice subjected to TR2 (*p* < 0.001) or TR10 (*p* < 0.001) sessions, the number of FosB and pCREB positive cells in the anterior portion of ACC was significantly lower compared to SDS mice in sedentary conditions (Fig 8A). While in the posterior portion of ACC, the number of pCREB positive cells was significantly lower in SDS mice subjected to TR2 and TR10 sessions (Fig 8B), TR2 and TR10 exerted less effects on FosB expressions in SDS mice (*p* > 0.05).

**Fig 9:**
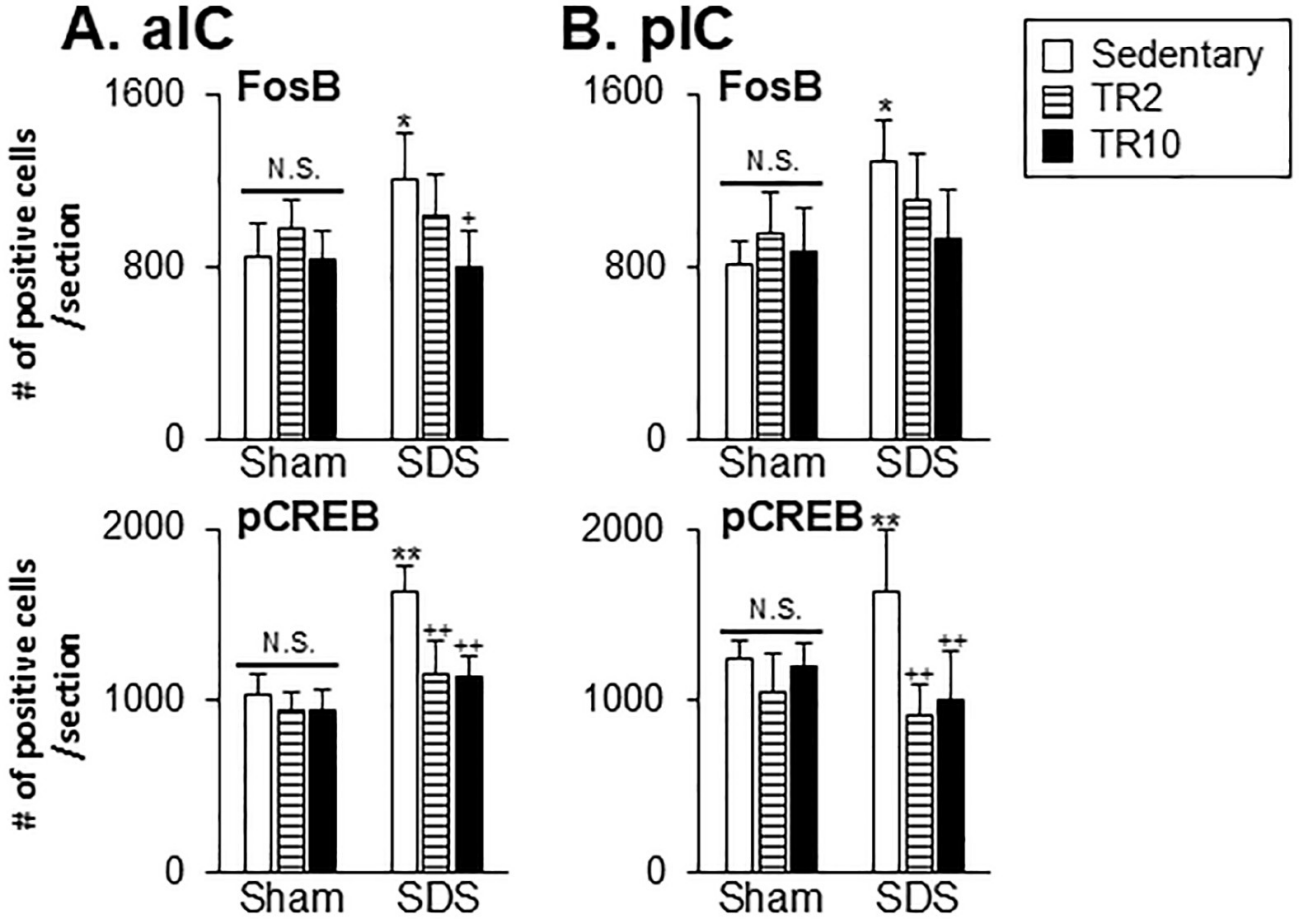
Data summary of the effect of treadmill running on FosB and pCREB expression in the anterior portion of IC (A; aIC) and posterior portion of IC (B; pIC). *, ** versus Sham–Sedentary group. +, ++ versus SDS-Sedentary group. **, ++ *p* < 0.001. *, + *p* <0.05. N.S. no significant when compared to the Sedentary group.

In the IC, under sedentary conditions, the number of FosB-positive (*p* < 0.05) and pCREB-positive (*p* < 0.001) cells were significantly higher in SDS mice compared to Sham mice (Fig 9).

In Sham mice, TR2 and TR10 exhibited less effect of FosB and pCREB expressions (*p* > 0.05) in the anterior portion of IC (Fig 9A) and posterior portion of IC (Fig 9B). The number of FosB-positive cells in the anterior portion of IC (*p* < 0.05), but not the posterior portion of IC (*p* > 0.05), was significantly lower in SDS mice subjected to TR10 compared to those in sedentary sessions. TR2 sessions had less effect on FosB expression in both the anterior (Fig 9A) and posterior portions of IC (Fig 9B).

In SDS mice subjected to TR2 and TR10, the number of pCREB-positive cells in both the anterior (*p* < 0.001) and posterior portions of IC (*p* < 0.001) was significantly lower compared to SDS mice in sedentary sessions.

#### Effect of TR on acetylated histone H3, HDAC1, and HDAC2 expression in the RVM and C2 regions

**Fig 10:**
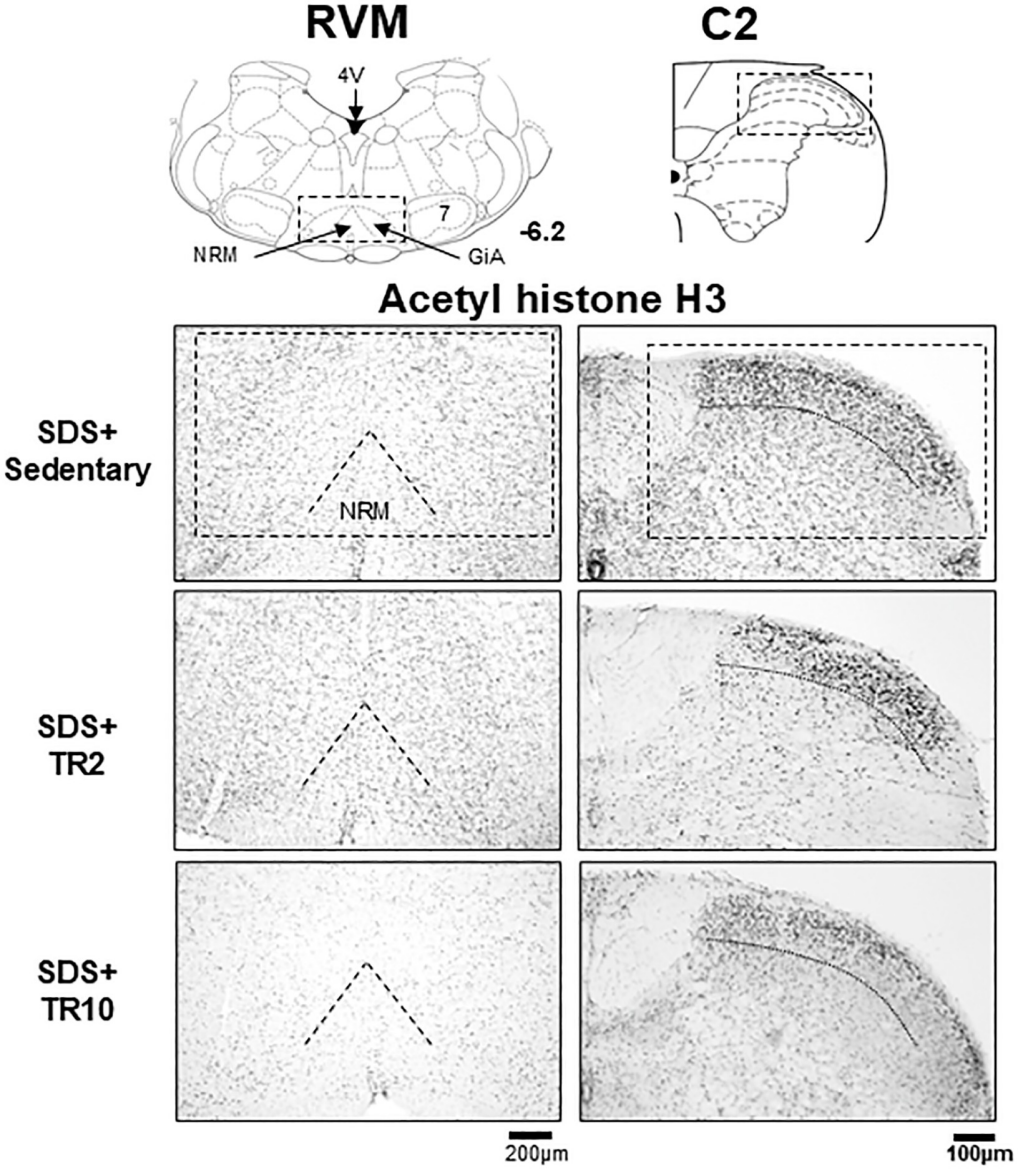
Microphotographs displaying acetylated histone H3 immunoreactivities in SDS mice in the RVM and C2 regions. TR10 sessions decreased acetylated (acetyl) histone H3 positive cells in RVM (left panel) but induced fewer changes in the C2 region (right panel). Cell counts were conducted within the boxed areas highlighted in each region, as illustrated in the brain schema shown in the top panels. The number below the left coronal section of the RVM indicates the rostrocaudal distance from the bregma (in mm). Abbreviations: 4V: the fourth ventricle; GiA: nucleus reticularis gigantocellularis pars alpha; NRM: nucleus raphe magnus; RVM: rostral ventromedial medulla; C2: upper cervical spinal cord.

Fig 10 illustrates examples of acetylated (acetyl) histone H3 immunoreactivities in SDS mice, focusing on the RVM and C2 regions. Similar to the ACC and IC regions (Fig 4), these immunoreactivities are predominantly localized in cell nuclei. In the C2 region (Fig 10), acetyl histone H3 immunoreactivities are primarily observed in the superficial lamina, whereas in the RVM (Fig 10), they appear more uniformly distributed.

Significant main effects of histone H3 acetylation expression were observed across six groups in the RVM region (F(5, 37) = 10.0, *p* < 0.0001) and the C2 region (F(5, 39) = 5.1, *p* < 0.001). Additionally, significant main effects were identified for HDAC1 expression in the C2 region (F(5, 33) = 9.5, *p* = 0.0001) and HDAC2 expression in the same region (F(5, 35) = 2.8, *p* = 0.033). In the RVM region, a marginal effect was observed for HDAC1 expression (F(5, 28) = 2.3, *p* = 0.063), while significant effects were noted for HDAC2 expression (F(5, 32) = 3.6, *p* < 0.01). Post hoc analysis using the Bonferroni test revealed the following.

**Fig 11:**
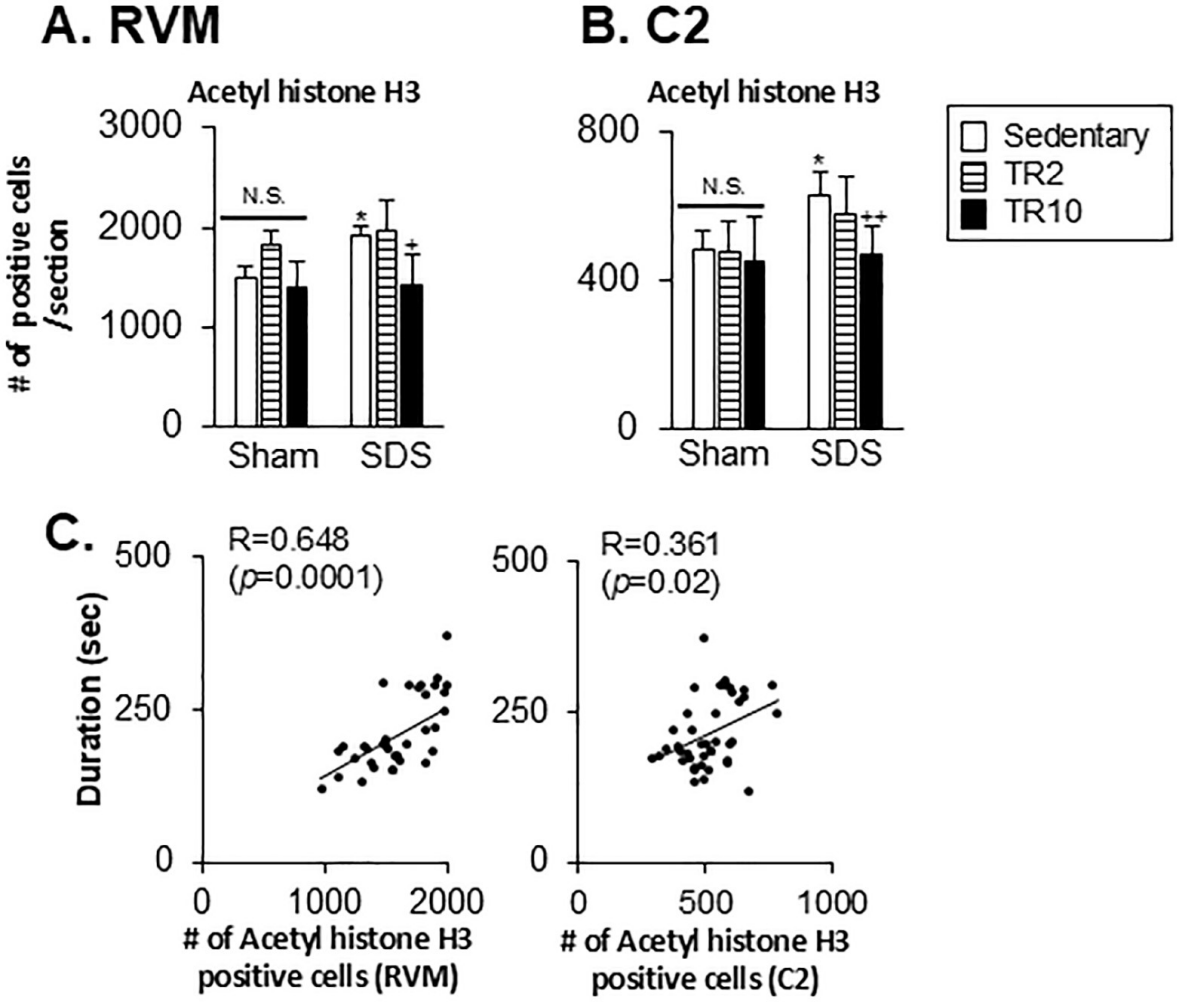
Data summary of the effect of treadmill running on acetylated (acetyl) histone H3 expression in the RVM (**A**) and C2 (**B**). (**C**) Scatter plots display the correlation between orofacial formalin behavior and acetyl histone H3 expression in the RVM and C2 regions. * *p* < 0.05 versus sham–Sedentary group, + *p* < 0.05, versus SDS–Sedentary group.

In Sham mice (Fig 11), neither TR2 nor TR10 exhibited a significant effect on acetyl histone H3 expressions in the RVM and C2 regions (*p* > 0.05). In SDS mice subjected to sedentary sessions, the number of acetyl histone H3 positive cells in the RVM (*p* < 0.05, Fig 11A) and C2 (*p* < 0.05, Fig 11B) regions were significantly greater than that in Sham mice subjected to sedentary sessions. In SDS mice subjected to TR10, the number of acetyl histone H3 positive cells in the RVM (*p* < 0.05, Fig 11A) and C2 (*p* < 0.001, Fig 11B) region was significantly lower compared to SDS mice under sedentary conditions. TR2 sessions exert less effect on them in SDS mice (*p* > 0.05).

Spearman’s test demonstrated that formalin-evoked craniofacial pain-like behaviors were positively correlated with H3 expression in RVM (r = 0.648, *p* = 0.0001) and C2 (r = 0.361, *p* = 0.02) (Fig 11C).

**Fig 12:**
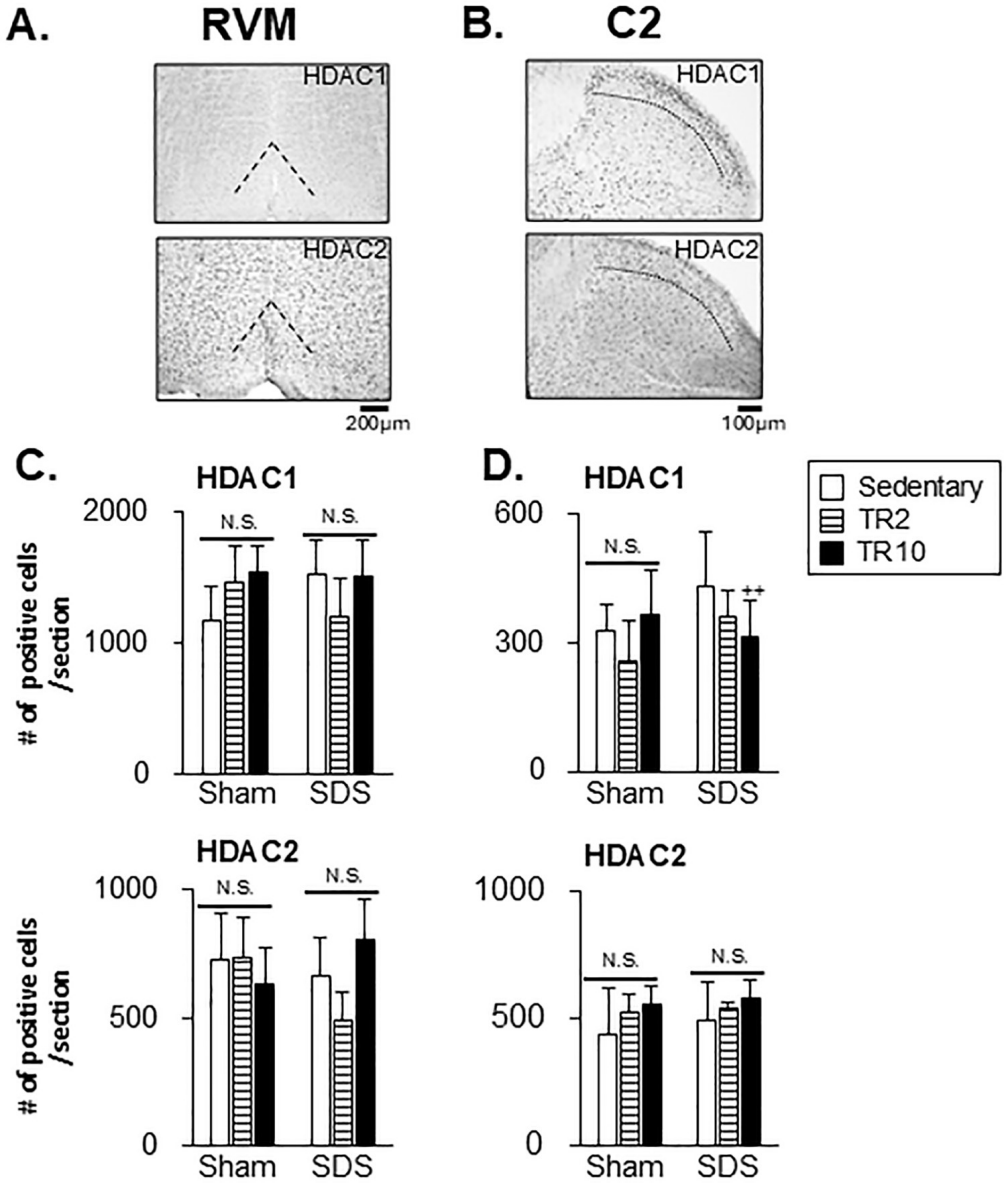
Effect of treadmill running on HDAC1 and HDAC2 expression in the RVM and C2 regions. (**A**) and (**B**) Examples of microphotographs displaying HDAC1 and HDAC2 immunoreactivities in SDS mice subjected to sedentary sessions in the RVM and C2 regions. (**C**) and (**D**) Data summary of the effect of treadmill running (TR) on HDAC1 and HDAC2 expression in the RVM (**C**) and C2 region (**D**). ++ *p* < 0.001versus SDS-Sedentary group. N.S. no significant when compared to the sedentary group.

Fig 12A and 12B illustrate examples of HDAC1 and HDAC2 immunoreactivity in the RVM and C2 regions from the SDS group. Both immunoreactivities are observed within cell nuclei. While HDAC1 and HDAC2 immunoreactivities in the RVM and HDAC2 in the C2 region appear consistent within the area evaluated, HDAC1 immunoreactivity in the C2 region seems stronger in the superficial compared to the deep laminae.

Fig 12C and 12D showed that the number of HDAC1 and HDAC2 in the RVM and C2 regions was not affected by SDS or TR. However, HDAC1 expression in the C2 region was significantly lower in SDS mice subjected to TR10 sessions compared to those that remained sedentary (*p* < 0.001, Fig 12D).

*Effect of TR on FosB and pCREB expression in the RVM and C2 regions*. Significant main effects were observed for the number of FosB and pCREB-positive cells across six groups in both the RVM (FosB: F(5, 36) = 3.3, *p* = 0.017; pCREB: F(5, 35) = 6.1, *p* < 0.0001) and C2 (FosB: F(5, 37) = 18.6, *p* < 0.0001; pCREB: F(5, 41) = 7.194, *p* < 0.0001) regions. Post hoc analysis using the Bonferroni test revealed the following.

**Fig 13:**
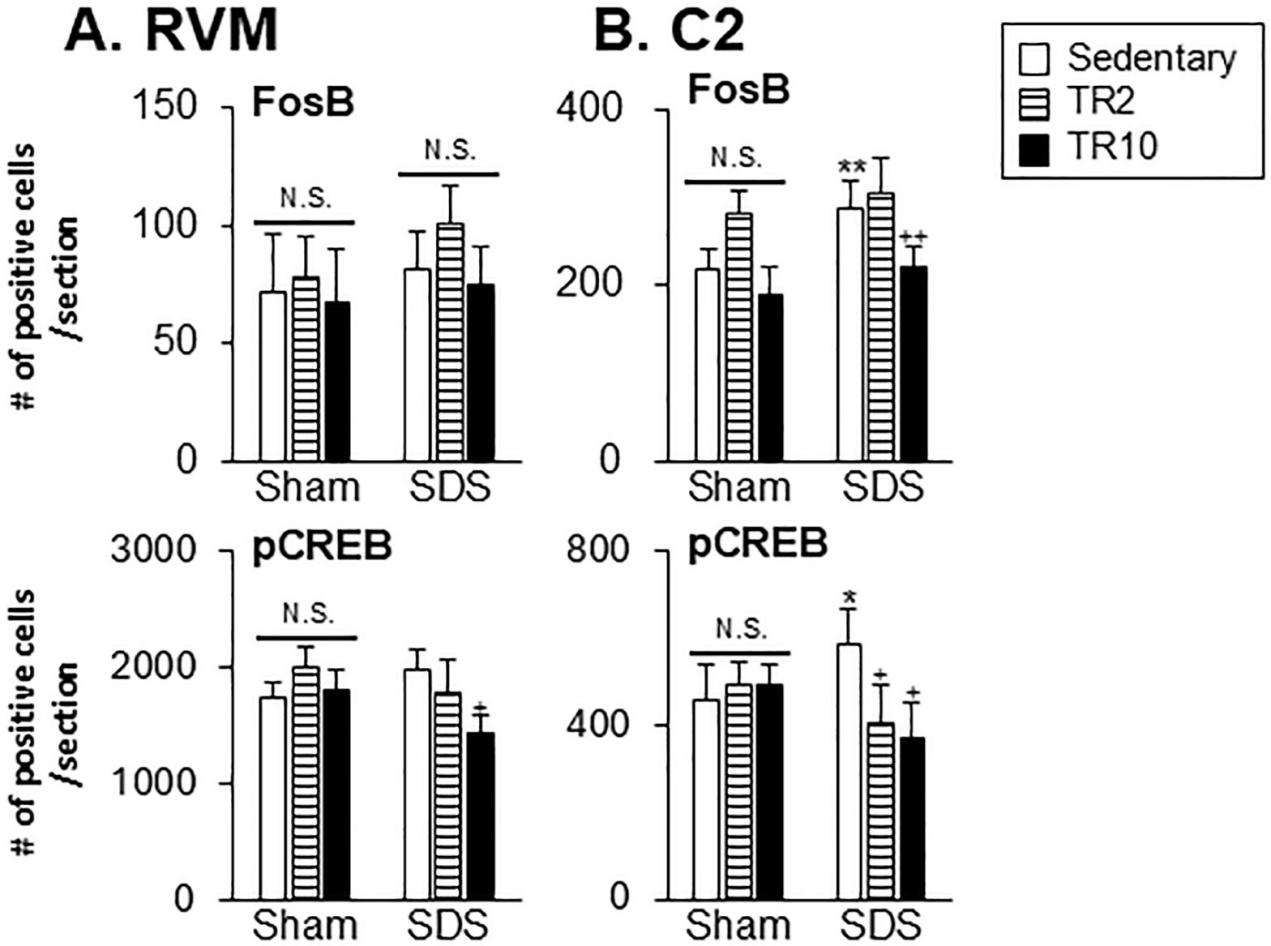
Data summary of the effect of treadmill running on FosB and pCREB expression in the RVM (A) and C2 (B). * *p* < 0.05, ** *p* < 0.001 versus Sham–Sedentary group, + *p* < 0.05, ++ *p* < 0.001 versus SDS-Sedentary group. N.S. no significant when compared to the sedentary group.

Fig 13A shows that the number of FosB and pCREB positive cells in the RVM of SDS mice subjected to sedentary sessions was similar to that of Sham mice in sedentary sessions (*p* > 0.05). In contrast, in the C2 region, the number of FosB (*p* < 0.001) and pCREB (*p* < 0.05) positive cells were significantly greater in SDS mice subjected to sedentary sessions compared to Sham mice under the same conditions (Fig 13B).

While TR2 and TR10 sessions did not affect FosB expression in the RVM of Sham and SDS mice (Fig 13A), TR10 sessions had a small but significant inhibitory effect on pCREB expression in the RVM of SDS mice (*p* < 0.05).

In the C2 region, the number of FosB and pCREB positive cells was significantly lower in SDS mice subjected to TR10 sessions compared to those in sedentary SDS mice (Fig 13B). However, the number of pCREB, but not FosB, positive cells was significantly lower in SDS mice subjected to TR2 sessions compared to those in sedentary SDS mice. TR2 and TR10 sessions had less effect on the number of FosB and pCREB-positive cells in Sham mice.

## Discussion

This study demonstrated the significant impact of treadmill running (TR) sessions in normalizing SDS-induced neural changes by preventing SDS-induced epigenetic changes in the brain and associated behavioral responses, including anxiety- and craniofacial pain-like behavior. In the following, we first briefly discuss the effects of SDS on epigenetic changes in the brain and then explore the preventive effects of TR under SDS conditions.

### Effects of SDS on epigenetic changes in the brain

Epigenetic modifications, particularly histone acetylation, play a critical role in stress-related disorders [19, 41]. Histone H3 acetylation, while reversible, can influence gene expression by altering chromatin structure, thereby impacting neural activity and behavior [14, 42, 43]. Our findings demonstrate that 10 days of SDS significantly increased histone H3 acetylation in brain cortices, including the ACC and IC. Similar findings are observed in the forced swim stress-induced pain model, demonstrating increased histone H3 acetylation in the brain cortices [38].

Our results demonstrated that increases in histone H3 acetylation induced by SDS were not limited to the ACC and IC but extended to the RVM and C2 regions. Neural changes in these regions, referred to as descending pain controls, could also increase craniofacial pain [1, 2, 6]. These findings are consistent with previous reports demonstrating increased histone H3 acetylation in the RVM [40] and the spinal lumbar dorsal horn [44, 45], which were observed in repeated restraint stress and various pain models, respectively, alongside enhanced pain-like responses.

Our SDS mice increased the expression levels of neural activity markers, FosB and pCREB, which were identified in the ACC, IC, RVM, and C2 regions. The interplay between these markers, histone H3 acetylation, and even the beneficial roles of TR on SDS-related behavioral responses remains unclear. However, changes in the level of marker proteins suggest that SDS causes functional changes in the brain, contributing to SDS-related craniofacial nociception.

Our study also determined the effects of SDS on the expression of histone deacetylase (HDAC) enzymes, which can suppress transcription by removing acetyl groups from histones. The role of HDACs in anxiety and chronic pain varies across specific HDAC classes, but changes in their levels might influence nociception and stress-related disorders [17, 27]. Our findings demonstrated that SDS increased HDAC1 levels in the ACC and IC, while HDAC2 levels were less affected. Additionally, SDS had a lesser impact on HDAC1 and HDAC2 expression in the RVM and C2 regions. These results suggest that SDS might regulate these enzymes differently depending on the areas in the brain and the enzyme class. The specific role of changes in histone H3 acetylation and HDAC levels in these brain areas on increased craniofacial nociception remains unclear; however, our results revealed that increased histone H3 acetylation in these regions was positively correlated with craniofacial pain-like behaviors.

### Effects of TR on anxiety- and craniofacial pain-like behaviors

TR10 decreased craniofacial pain- and anxiety-like behaviors in SDS mice. These results align with prior findings indicating that prolonged TR regimens (> 10 days) are essential for reducing pain- and anxiety-like responses in various pain and chronic stress models [7, 16, 46, 47]. This inhibitory effect on increased formalin-evoked craniofacial pain-like behaviors, particularly in the late phase but not the early phase, suggests that TR10 might influence neural function in the brain. This effect might potentially involve the normalization of neural changes, such as epigenetic changes because late-phase formalin-evoked pain-like behaviors are known to be associated with functional changes in the central nervous system rather than the peripheral nervous system [48].

In sham mice, TR10 had a less inhibitory effect on anxiety- and craniofacial pain-like behaviors. Indeed, TR10 significantly increased time spent within the center areas and total movement distance in the OF (Fig 1B and 1C). However, variations in TR parameters might contribute to these differences in outcomes, as mechanical hyperalgesia-like behaviors were significantly reduced by TR sessions conducted before the induction of knee joint inflammation [49]. Indeed, the parameters of TR (10 m/min, 60 min/day, 30 days) employed in the report [49] were different from ours (6 m/min, 30 min/day, 10 days).

A shorter 2-day treadmill running (TR2) regimen did not reduce craniofacial pain-like behaviors in SDS mice. This is consistent with previous findings that TR sessions conducted every three days over a 10-day SDS period (three sessions total) similarly failed to alter facial pain-like behaviors [7]. However, our findings show that TR2 reduced anxiety-like behaviors in SDS mice, as demonstrated across multiple tests, including the OF and EPM procedures. It is speculated that the neural mechanisms underlying SDS-induced anxiety-like responses might be different from those for craniofacial nociception, at least partially, in their sensitivity to TR sessions.

Unexpectedly, under sham conditions, TR2, but not TR10 increased formalin-evoked craniofacial pain-like behaviors in the early phase. This suggests neural changes in the central nervous system are unlikely because the early phase of pain-like behaviors is known to be associated with neural changes in the periphery [48]. Accordingly, it is speculated that peripheral sensitization might be potentially linked to inflammation in the body, including muscles of limbs, caused by 2-day TR before behavioral tests. Subsequently, acute hyperalgesia-like responses after TR2 might also explain decreased movement distance in the OF test (Fig 2C). Increased FosB expression in the anterior portion of ACC (Fig 8A) supports these findings. Furthermore, TR2 also increased anxiety-like behaviors identified by the OF and SI tests, which is consistent with previous reports of anxiety-like effects after single TR sessions [50].

### Effects of TR on epigenetic changes in the brain

Changes in histone H3 acetylation levels in the brain have been implicated in psychological stress-induced behavioral changes [19, 27, 51]. Our findings revealed the involvement of epigenetic mechanisms in the brain in mediating the effects of TR on SDS-induced anxiety- and craniofacial pain-like behaviors. The impact of TR on histone H3 acetylation varies across different brain regions. For example, daily TR for four weeks increased histone H3 expression in the motor cortex [52], and a two-week TR regimen normalized decreases in hippocampal histone H3 acetylation and anxiety-like behaviors induced by SDS [14].

TR10 prevented SDS-induced increases in histone H3 acetylation expression in the ACC and IC, areas implicated in anxiety and nociceptive processing. Interestingly, while the effects were more pronounced in the anterior portions of these regions, consistent with their roles in emotional and nociception [37, 53–57]. This effect was less pronounced in the posterior portion of the IC. These findings indicated that TR10 modulates SDS-induced epigenetic changes in a region-specific manner within the ACC and IC. While increases in c-Fos and FosB expression in the anterior portion of the ACC and IC following a 10-day SDS were positively correlated with craniofacial pain-like behaviors [5], our results revealed that TR10 could normalize the FosB and pCREB expression in the anterior portion of the both ACC and IC.

The lack of significant effects of TR2 on histone H3 acetylation aligns with its less impact on craniofacial pain-like behaviors under SDS conditions. However, TR2 decreased pCREB expression in both the anterior and posterior portions of the ACC and IC, as well as FosB expression in the anterior portion of the ACC. While the precise interpretation of these distinct regulatory effects remains unclear, TR2 might have distinct roles on these markers that underlie the distinct behavioral effects of craniofacial nociception.

Evidence suggests that TR sessions can prevent neural changes in the RVM and spinal dorsal horn by normalizing various molecular mechanisms, including intracellular signaling and opioid, serotonergic, and glutamatergic systems, reducing pain-like responses [11, 16]. Our findings showed that under SDS conditions, TR10 could normalize the histone H3 acetylation, FosB, and pCREB expressions in the RVM and C2 regions. While the precise link between histone H3 acetylation and these molecular changes remains unclear, it is reasonable to mention that TR10 could at least exert regulatory roles on neural functions in the RVM and C2 regions.

In contrast, TR2 had less effect on histone H3 acetylation expression in these regions. These findings align with less alteration in the level of FosB and pCREB expression in the RVM and FosB in the C2 region. Hence, a short-term TR regimen seems to exert minor roles on the neural function in these areas compared with the findings seen in the ACC and IC.

One intriguing aspect of our findings is the paradoxical relationship between HDACs and histone H3 acetylation. Both TR2 and TR10 decreased HDAC1 expression in the ACC and IC under SDS conditions, yet TR10 reduced histone H3 acetylation levels. This is unexpected, as HDAC1 typically inhibits histone H3 acetylation. A potential explanation is that TR10 might regulate histone H3 acetylation through alternative pathways independent of HDAC1 activity. TR10 might modulate the activity of other histone deacetylases or histone acetyltransferases, resulting in a net reduction in acetylation [14, 42, 43]. Future studies should evaluate these alternative pathways to elucidate the mechanisms underlying this paradox.

In contrast to the findings observed in the ACC and IC, neither SDS nor TR changed the expression levels of HDAC1 and HDAC2 in the RVM or C2 region; however, in a bone cancer pain model, HDAC1 and HDAC2 levels were significantly increased in the spinal cord, and inhibition of their enzymatic activity effectively reduced hyperalgesia-like behaviors in the hindpaw [58]. This discrepancy might be due to the differences in methodologies, including animal models. In the case of the bone cancer pain model, the level of enzymes seen in the spinal dorsal horn is prominently influenced by the environmental changes from the bone cancer. However, this is not the case for the SDS model.

## Conclusion

Our findings demonstrated that a daily TR regimen (6 m/min, 30 minutes/day) over 10 days could normalize increased anxiety-like and craniofacial pain-like behaviors induced by a 10-day SDS protocol. The 10-day TR regimen (TR10) also reversed SDS-related epigenetic changes, as evidenced by normalized histone H3 acetylation levels. In contrast, a shorter-term TR regimen (TR2) failed to produce these effects. These results suggest that a prolonged TR regimen might represent an option for a non-pharmacological approach to managing chronic craniofacial pain conditions.

## Supporting information

S1 File List of sample size. (DOCX)

S2 File Excel data of the experiments. (ZIP)
