# Supplementary material for: Effects of treadmill running on anxiety- and craniofacial pain-like behaviors with histone H3 acetylation in the brain of mice subjected to social defeat stress
Source: PLoS One. 2025 Jan 27;20(1):e0318292. doi: 10.1371/journal.pone.0318292 (PMC11771924; doi:10.1371/journal.pone.0318292)
Supplement: S1 File — (DOCX) [file pone.0318292.s001.docx]

**S1. The sample size in each experiment.**

**1. Anxiety-like behavior demonstrated in Fig 2.**

OF test (**B**, **C**): Sham–Sedentary (n = 13), Sham–TR2 (n = 8), Sham–TR10 (n = 11), SDS–Sedentary (n = 10), SDS–TR2 (n = 12), SDS–TR10 (n = 9).

EPM test (**E**): Sham–Sedentary (n = 10), Sham–TR2 (n = 8), Sham–TR10 (n = 8), SDS–Sedentary (n = 10), SDS–TR2 (n = 12), SDS–TR10 (n = 9).

DL test (**F**): Sham–Sedentary (n = 16), Sham–TR2 (n = 8), Sham–TR10 (n = 16), SDS–Sedentary (n = 10), SDS–TR2 (n = 12), SDS–TR10 (n = 9).

SI test (**H**): Sham–Sedentary (n = 13), Sham–TR2 (n = 8), Sham–TR10 (n = 8), SDS–Sedentary (n = 10), SDS–TR2 (n = 12), SDS–TR10 (n = 9).

**2. Orofacial formalin test demonstrated in Fig 3.**

Sham–Sedentary (n = 11), Sham–TR2 (n = 9), Sham–TR10 (n = 8), SDS–Sedentary (n = 13), SDS–TR2 (n = 12), SDS–TR10 (n = 13).

**3. Immunohistochemistry for histone H3 acetylation, HDAC1 and HDAC2 shown in Figs 6A and 6B**.

aACC/acetylated histone H3: Sham–Sedentary (n = 7), Sham–TR2 (n = 5), Sham–TR10 (n = 8), SDS–sedentary (n = 11), SDS–TR2 (n = 7), SDS–TR10 (n = 10).

aACC/HDAC1: Sham–Sedentary (n = 5), Sham–TR2 (n = 5), Sham–TR10 (n = 5), SDS–Sedentary (n = 5), SDS–TR2 (n = 5), SDS–TR10 (n = 6).

aACC/HDAC2: Sham–Sedentary (n = 6), Sham–TR2 (n= 7), Sham–TR10 (n= 8), SDS–Sedentary (n = 8), SDS–TR2 (n = 9), SDS–TR10 (n = 6).

pACC/acetylated histone H3: Sham–Sedentary (n = 7), Sham–TR2 (n = 5), Sham–TR10 (n = 8), SDS–sedentary (n = 11), SDS–TR2 (n = 8), SDS–TR10 (n = 10).

pACC/HDAC1: Sham–Sedentary (n = 6), Sham–TR2 (n = 6), Sham–TR10 (n = 6), SDS–Sedentary (n = 6), SDS–TR2 (n = 6), SDS–TR10 (n = 6).

pACC/HDAC2: Sham–Sedentary (n = 6); Sham–TR2 (n = 7), Sham–TR10 (n = 8); SDS–Sedentary (n = 8), SDS–TR2 (n = 9); SDS–TR10 (n = 6).

**4. Spearman’s test shown in Fig 6C**.

aACC: Sham–Sedentary (n = 7), Sham–TR2 (n = 5), Sham–TR10 (n = 8), SDS–Sedentary (n = 10), SDS–TR2 (n = 6), SDS–TR10 (n = 10).

pACC: Sham–Sedentary (n = 7), Sham–TR2 (n = 5), Sham–TR10 (n = 7), SDS–Sedentary (n = 10), SDS–TR2 (n = 9), SDS–TR10 (n = 7).

**5. Immunohistochemistry for histone H3 acetylation, HDAC1 and HDAC2 in the IC shown in Figs 7A and 7B**.

aIC/acetylated histone H3: Sham–Sedentary (n = 7), Sham–TR2 (n = 6), Sham–TR10 (n = 8), SDS–sedentary (n = 10), SDS–TR2 (n = 6), SDS–TR10 (n = 8).

aIC/HDAC1: Sham–Sedentary (n = 6), Sham–TR2 (n = 6), Sham–TR10 (n = 6), SDS–Sedentary (n = 6), SDS–TR2 (n = 6), SDS–TR10 (n= 5).

aIC/HDAC2: Sham–Sedentary (n = 6), Sham–TR2 (n = 7), Sham–TR10 (n = 8), SDS–Sedentary (n = 6), SDS–TR2 (n = 7), SDS–TR10 (n = 8).

pIC/acetylated histone H3: Sham–Sedentary (n = 7), Sham–TR2 (n = 6), Sham–TR10 (n = 8), SDS–sedentary (n = 13), SDS–TR2 (n = 6), SDS–TR10 (n = 8).

pIC/HDAC1: Sham–Sedentary (n = 6), Sham–TR2 (n = 6), Sham–TR10 (n = 6), SDS–Sedentary (n = 6), SDS–TR2 (n = 6), SDS–TR10 (n= 6).

pIC/HDAC2: Sham–Sedentary (n = 8); Sham–TR2 (n = 9), Sham–TR10 (n = 6); SDS–Sedentary (n= 8), SDS–TR2 (n= 9); SDS–TR10 (n= 6).

Sample sizes were analyzed in Spearman’s test.

aIC: Sham–Sedentary (n = 7), Sham–TR2 (n = 5), Sham–TR10 (n = 8), SDS–Sedentary (n = 10), SDS–TR2 (n = 6), SDS–TR10 (n = 8).

pIC: Sham–Sedentary (n = 7), Sham–TR2 (n = 5), Sham–TR10 (n = 7), SDS–Sedentary (n = 10), SDS–TR2 (n = 6), SDS–TR10 (n = 7).

**6. Spearman’s test shown in Fig 7C**.

aIC: Sham–Sedentary (n = 7), Sham–TR2 (n = 5), Sham–TR10 (n = 8), SDS–Sedentary (n = 10), SDS–TR2 (n = 6), SDS–TR10 (n = 8).

pIC: Sham–Sedentary (n = 7), Sham–TR2 (n = 5), Sham–TR10 (n = 7), SDS–Sedentary (n = 10), SDS–TR2 (n = 6), SDS–TR10 (n = 7).

**7. Immunohistochemistry for FosB and pCREB shown in Fig 8**.

aACC/FosB: Sham–Sedentary (n = 8), Sham–TR2 (n = 5), Sham–TR10 (n = 8), SDS–Sedentary (n = 11), SDS–TR2 (n = 9), SDS–TR10 (n = 10).

pACC/FosB: Sham–Sedentary (n = 8), Sham–TR2 (n = 5), Sham–TR10 (n = 8), SDS–Sedentary (n = 11), SDS–TR2 (n = 9), SDS–TR10 (n = 9).

aACC/pCREB: Sham–Sedentary (n = 6), Sham–TR2 (n = 7), Sham–TR10 (n =8), SDS–sedentary (n = 7), SDS–TR2 (n = 8), SDS–TR10 (n = 6).

pACC/pCREB: Sham–Sedentary (n = 6), Sham–TR2 (n = 7), Sham–TR10 (n = 9), SDS–Sedentary (n = 7), SDS–TR2 (n = 8), SDS–TR10 (n = 6).

**8. Immunohistochemistry for FosB and pCREB shown in Fig 9**.

aIC/FosB: Sham–Sedentary (n = 6), Sham–TR2 (n = 7), Sham–TR10 (n =7), SDS–Sedentary (n = 8), SDS–TR2 (n = 8), SDS–TR10 (n = 9).

pIC/FosB: Sham–Sedentary (n = 6), Sham–TR2 (n = 7), Sham–TR10 (n = 7), SDS–Sedentary (n = 8), SDS–TR2 (n = 8), SDS–TR10 (n = 9).

aIC/pCREB: Sham–Sedentary (n = 6), Sham–TR2 (n = 7), Sham–TR10 (n = 8), SDS–sedentary (n = 6), SDS–TR2 (n = 7), SDS–TR10 (n = 8).

pIC/pCREB: Sham–Sedentary (n = 8), Sham–TR2 (n = 9), Sham–TR10 (n = 6), SDS–Sedentary (n = 8), SDS–TR2 (n = 8), SDS–TR10 (n = 6).

**9. Immunohistochemistry for histone H3 acetylation shown in Figs 11A and 11B**.

RVM: Sham–Sedentary (n = 6), Sham–TR2 (n = 5), Sham–TR10 (n = 9), SDS–Sedentary (n = 7), SDS–TR2 (n = 8), SDS–TR10 (n = 8).

C2: Sham–Sedentary (n = 6), Sham–TR2 (n = 5), Sham–TR10 (n = 9), SDS–sedentary (n = 8), SDS–TR2 (n = 8), SDS–TR10 (n = 9).

**10.** **Spearman’s test shown in Fig 11C**.

RVM: Sham–Sedentary (n = 6), Sham–TR2 (n = 4), Sham–TR10 (n = 8); SDS–sedentary (n = 7), SDS–TR2 (n =7), SDS–TR10 (n = 7).

C2: Sham–Sedentary (n = 6), Sham–TR2 (n = 4), Sham–TR10 (n = 8), SDS–Sedentary (n = 8), SDS–TR2 (n = 6), SDS–TR10 (n = 9).

**11. Immunohistochemistry for HDAC1 and HDAC2 shown in Figs 12C and 12D**.

RVM/HDAC1: Sham–Sedentary (n= 5), Sham–TR2 (n= 5), Sham–TR10 (n= 6), SDS–Sedentary (n= 4), SDS–TR2 (n= 8), SDS–TR10 (n= 6).

RVM/HDAC2: Sham–Sedentary (n= 6); Sham–TR2 (n= 6); Sham–TR10 (n= 8), SDS–Sedentary (n= 5), SDS–TR2 (n= 7), SDS–TR10 (n= 6).

C2/HDAC1: Sham–Sedentary (n= 6); Sham–TR2 (n= 7), Sham–TR10 (n= 6), SDS–Sedentary (n= 7), SDS–TR2 (n= 7), SDS–TR10 (n= 6).

C2/HDAC2: Sham–Sedentary (n= 5), Sham–TR2(n = 7), Sham–TR10 (n= 7), SDS–Sedentary (n= 8), SDS–TR2 (n= 8), SDS–TR10 (n= 6).

**12. Immunohistochemistry for FosB and C2 shown in Figs 13A and 13B**.

**RVM/FosB**: Sham–Sedentary (n = 6), Sham–TR2 (n = 6), Sham–TR10 (n = 8), SDS–Sedentary (n = 8), SDS–TR2 (n = 9), SDS–TR10 (n = 6).

**RVM/pCREB**: Sham–Sedentary (n = 6); Sham–TR2 (n = 6), Sham–TR10 (n = 8), SDS–Sedentary (n = 7), SDS–TR2 (n = 9), SDS–TR10 (n = 5).

**C2/FosB**: Sham–Sedentary (n = 6), Sham–TR2 (n = 6), Sham–TR10 (n = 8), SDS–Sedentary (n = 8), SDS–TR2 (n = 9), SDS–TR10 (n = 6).

**C2/pCREB**: Sham–Sedentary (n = 6), Sham–TR2 (n = 5), Sham–TR10 (n = 8), SDS–Sedentary (n = 8), SDS–TR2 (n = 9), SDS–TR10 (n = 6).
